# Insight into the Interaction Mechanism of Vitamin D against Metabolic Syndrome: A Meta-Analysis and In Silico Study

**DOI:** 10.3390/foods12213973

**Published:** 2023-10-30

**Authors:** Yuting Xia, Yuandong Yu, Yi Zhao, Zhifen Deng, Lei Zhang, Guizhao Liang

**Affiliations:** 1 Key Laboratory of Biorheological Science and Technology, Ministry of Education, Bioengineering College, Chongqing University, Chongqing 400044, China; 202219021088t@stu.cqu.edu.cn (Y.X.); yuandongncm@163.com (Y.Y.); 20221901021@stu.cqu.edu.cn (Y.Z.); 202119021155t@cqu.edu.cn (Z.D.); 2 College of Life Science, Chongqing Normal University, Chongqing 401331, China

**Keywords:** vitamin D, lipid profile, insulin resistance, metabolic syndrome, meta-analysis, network pharmacology, molecular dynamics simulation

## Abstract

**Highlights:**

**What are the main findings?**

- In total, 14 core genes for vitamin D against MetS are obtained via enrichment analysis.
- Van der Waals forces are the main interactions to sustain vitamin D binding with targets.

**What is the implication of the main finding?**

- Vitamin D could reduce triglycerides significantly and improve insulin resistance.
- Vitamin D might modulate the PPAR signaling pathway and inhibit bile secretion.

**Abstract:**

As a dietary supplement or functional food additive, vitamin D (VD) deficiency may impact extra-skeletal functions associated with metabolic syndrome (MetS) risk factors. However, the precise effects and mechanisms of VD supplementation on dyslipidemia and insulin resistance in MetS subjects remain controversial. Here, we investigate potential therapeutic targets, pathways and mechanisms of VD against MetS through a comprehensive strategy including meta-analysis, network pharmacology analysis, molecular docking, dynamics simulations, and quantum chemical calculations. Our results reveal that VD supplementation significantly reduces triglyceride levels, fasting glucose, and insulin concentrations in subjects, thereby improving insulin homeostasis to some extent. We theoretically identify 14 core MetS-associated targets. Notably, VD exhibits substantial interactions with three targets (PPARγ, FABP4, and HMGCR) in the PPAR signaling pathway, indicating that VD can modulate this pathway. Van der Waals forces predominantly stabilize the complexes formed between VD and the three targets. Nonetheless, to provide valuable insights for personalized MetS management, further research is necessary to confirm our findings, emphasizing the importance of exploring genetic variability in VD response. In conclusion, our study contributes insights into the mechanisms of VD in preventing and treating MetS through dietary supplementation, promoting the development of VD-based functional foods or nutritious diets.

## 1. Introduction

Metabolic syndrome (MetS) entails a clinical constellation of physiological and biochemical metabolic disorders, encompassing abnormal lipid profiles, insulin resistance, and central obesity and serving as a risk factor for cardiovascular disease (CVD) [1]. According to official data from Amnesty International, the nationwide death toll due to CVD was reported at 18.56 million, with China alone accounting for 4.58 million CVD-related deaths [2]. Although statins are widely recognized as a crucial drug class for treating MetS, they are often accompanied by adverse effects such as hepatotoxicity, nephrotoxicity, and rhabdomyolysis [3].

Hence, it is imperative to consider preventive measures for MetS, such as dietary supplements, to mitigate the side effects of drug therapy and enhance the overall quality of life for patients. Vitamin D (VD) is well-established for its multifaceted applications in the realm of nutrition, primarily through food fortification or supplementation, aimed at increasing VD accessibility, especially for individuals with limited sun exposure or dietary restrictions [4,5]. As we all known, VD consists of two primary forms, ergocalciferol (VD_2_) and cholecalciferol (VD_3_), and can be activated through both canonical and non-canonical pathways [6,7]. In the canonical pathway, VD undergoes sequential hydroxylation at C-25α and C-1α by the enzymes CYP27A1 and CYP27B1, respectively. In addition, VD is hydroxylated by CYP11A1 in the emerging non-canonical pathway [8]. While the phenotypic activity of active forms of VD is predominantly mediated through the vitamin D receptor (VDR) [6,9], they can also regulate phenotype through alternative nuclear receptors, including retinoic acid receptor-related orphan receptor alpha/gamma (ROR α/γ) and liver X receptor alpha/beta (LXR α/β) [10]. Given that ROR α/γ and LXR α/β play critical regulatory roles in lipid metabolism, it is plausible that VD may be associated with the risk of chronic diseases such as MetS, CVD, and diabetes [11]. Consequently, VD supplementation and VD-fortified foods may help reduce the risk of these diseases. Recent studies have shown that VD supplementation could have beneficial effects on MetS biomarkers, such as improved arterial stiffness, increased peripheral insulin sensitivity, and reduced inflammatory cytokines [12,13]. However, conflicting findings exist, as some studies have reported that VD supplementation increased high-density lipoprotein cholesterol (HDL-C) in CVD patients but did not significantly impact other lipid profile markers such as total cholesterol (TC), low-density lipoprotein cholesterol (LDL-C), and triglycerides (TGs) [14]. Moreover, certain trials found no significant influence of VD supplementation on lipid profiles, inflammation biomarkers, or insulin resistance in pre-diabetic patients [15,16]. While animal experiments [17] and observational studies [18] have suggested some degree of improvement in MetS pathology with VD supplementation, the mechanism underlying the relationship between VD and MetS biomarkers remains controversial, primarily due to the lack of conclusive scientific evidence [19].

Therefore, more relevant randomized controlled trials (RCTs) are required to substantiate the efficacy of VD supplementation in preventing and treating MetS. To address this, we employed a meta-analysis approach, which is a statistically rigorous method involving the systematic review and quantitative synthesis of data from previous studies. Meta-analysis provides notable advantages, including cost-effectiveness and feasibility compared to large-scale trials. This approach has gained extensive use in food science, particularly in investigating prevention and treatment strategies for cardiovascular diseases [20,21,22,23,24,25]. Furthermore, network pharmacology, as a methodology that reevaluates existing drugs, aids in discovering the potential efficacy and mechanisms of drugs already approved for treating new diseases, thus reducing the time and cost of developing new drugs [26,27,28,29,30]. In addition, systematic network pharmacology has been applied in the food field to elucidate the mechanisms underlying the interactions between small molecules and diseases [31,32]. In summary, meta-analysis comprehensively estimates overall effects by consolidating findings from multiple independent studies. The incorporation of network pharmacology further enables the synthesis of intricate relationships between drugs and diverse biomolecules, encompassing targets, pathways, and biological effects, thus furnishing a more exhaustive body of evidence [33,34,35].

**Figure 1:**
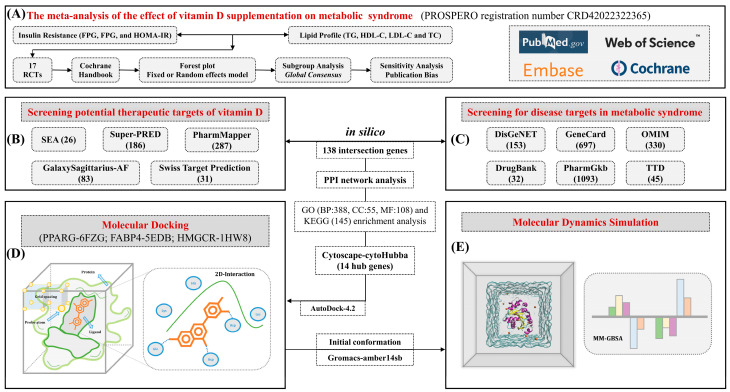
The research flow chart of the meta-analysis and in silico approach. (**A**) The primary research outcomes of the meta-analysis included lipid profiles and insulin resistance. (**B**,**C**) Screening potential targets for VD against MetS through in silico methods. (**D**,**E**) Obtaining initial conformations of the three core target proteins with VD through molecular docking, followed by molecular dynamics simulations to explore the interactions between the proteins and VD.

Here, we conducted a meta-analysis and employed network pharmacology to explore the potential pharmacological mechanisms and identify functional targets of VD in treating MetS. Furthermore, molecular docking and dynamics simulations were utilized to elucidate the interactions between VD and core targets. This study aims to provide valuable insights into dietary micronutrient supplementation, mainly focusing on VD as a strategy against MetS. In addition, we present a visual representation of our research process to illustrate the anti-MetS effects and mechanisms of VD (Figure 1).

## 2. Materials and Methods

### 2.1. Search Strategy Based on Inclusion and Exclusion Criteria

Inclusion and exclusion criteria for this study are described in Supplementary Materials Section S1.1. A systematic literature search was conducted across various databases, including PubMed, Web of Science, Embase, Cochrane Library, Ovid, Scopus, and ProQuest. We used a combination of keywords such as ‘Metabolic syndrome’, ‘Vitamin D supplements’, ‘Lipids’, ‘Cholesterol’, ‘Triglycerides’, ‘Cholecalciferol’, ‘Ergocalciferol’, and related medical subject headings (MeSH) terms to identify studies on VD supplementation in subjects with MetS. We searched for RCTs involving subjects with MetS from the inception of the databases up to July 2023. Furthermore, RCTs meeting the inclusion criteria were also gathered from relevant reviews to minimize potential bias associated with excluding gray literature. The search strategy of PubMed is outlined in Table S1.

### 2.2. Data Extraction and Bias Assessment of Individual Studies

From each study, the following information was obtained:

(1) Basic details about the included studies, including the literature title, first author, and publication year. (2) Key characteristics of the study population, such as age, sample size, serum 25(OH)D levels, missed follow-up rate, and duration of follow-up. (3) Details of the VD intervention, including dosage and form. (4) Measurement data for baseline and endpoint lipid- or glucose-related outcome indicators.

In addition, the risk of bias (Table S2) was independently evaluated by investigators for the quality of the included RCTs, following the guidelines outlined in the *Cochrane Handbook (version 5.1.0)*. When disagreements arose, an instructor assisted in the adjudication process.

### 2.3. Meta-Analysis of Included RCTs

If the extracted data were not normally distributed, we employed the methods described by Wan et al. [36] and Luo et al. [37] to process the data and obtain the appropriate mean difference (*MD*) and standard deviation (*SD*). Then, we calculated the *MD* and *SD* for the pre- and post-intervention change values of outcome indicators, including lipid profile and insulin resistance. The unit conversions for all indicators are shown in Table S3.

(1) $$MD=Mean\left(endpoint\right)-Mean\left(baseline\right)$$

(2) $$SD=\sqrt[{2}]{{SD}^{2}\left(baseline\right)+{SD}^{2}\left(endpoint\right)-2\times 0.5\times SD\left(baseline\right)\times SD\left(endpoint\right)}$$

The forest plots and sensitivity analysis were performed using *Review Manager 5.4*. We explored the sources of heterogeneity in the effect on lipid profile by performing subgroup analysis based on subjects’ baseline serum 25(OH)D levels.

### 2.4. In Silico Identification of VD Anti-MetS Targets

Preliminary MeSH terms of disease were collected from the PubMed database. For retrieval keywords, we used MeSH terms such as ‘metabolic syndrome’, ‘dyslipidemia’, and ‘insulin resistance’. In addition, the MetS-driven targets and all predicted targets of VD were obtained from relevant databases (Table S4) [38,39,40,41,42,43,44,45,46,47,48]. To convert protein names to gene symbols, we utilized the Retrieve/ID mapping function in the UniProt database (https://www.uniprot.org/) and selected the species as ‘human’.

### 2.5. Screening of Intersection Genes and Hub Genes

We calculated the intersection of the two target sets to elucidate the interaction between VD-related targets and disease-associated targets. Subsequently, we created a Venn diagram using the R programming language. Then, the intersecting targets were submitted to the STRING 11.0 database (https://string-db.org/) to construct the protein–protein interaction (PPI) network. The PPI network was constructed by setting the organism species as ‘Homo sapiens’, the minimum interaction threshold as ‘highest confidence’ (>0.9), and the rest of the settings as default. We employed the cytoHubba 0.1 plugin in *Cytoscape (dev3.9.1)* to screen the hub genes. The top 10 hub genes were screened using 12 different algorithms to create a heat map. In addition, we consulted the NCBI database (https://www.ncbi.nlm.nih.gov/) to obtain the significant roles of hub genes in metabolic regulation and to further screen for targets associated with MetS.

### 2.6. Identifying Key Pathways through GO and KEGG Enrichment Analysis

The intersecting genes were enriched and analyzed using the DAVID (http://david.abcc.ncifcrf.gov/). To recalibrate the false discovery rate (FDR) arising from the multiple hypothesis testing, we employed the Benjamini–Hochberg method (*p* < 0.05 indicated significance) [49]. Then, biological processes, cell components, and molecular functions associated with MetS according to FDR for Gene Ontology (GO) annotation and Kyoto Encyclopedia of Genes and Genomes (KEGG) pathway were screened for enrichment analysis. The GO chord diagram of core genes was plotted using Bioinformatics tools (http://www.bioinformatics.com.cn/), and KEGG pathway annotation was performed using OmicShare tools (https://www.omicshare.com/tools/, accessed on 1 October 2022). Both of these platforms are online tools for data analysis and visualization.

### 2.7. Molecular Docking

The receptor proteins regulated by the core genes were downloaded from the RCSB-PDB database (https://www.rcsb.org/). The PubChem database (https://pubchem.ncbi.nlm.nih.gov/) was searched to obtain the 3D molecular structure formulae (in SDF format) for VD_2_ (ergocalciferol, CID 5280793) and VD_3_ (cholecalciferol, CID 5280795). The LibDock mode in *Discovery Studio 2019* was utilized for 10 docking runs under the CHARMM force field. If the root-mean-square deviation (RMSD) ≤ 2 Å, it indicates a better reproduction of the binding pattern, rendering the docking results more reliable [50].

### 2.8. Molecular Dynamics (MD) Simulations

MD simulations were conducted using *GROMACS (version 2023)* with the AMBER FF14SB force field, as described in previous studies [51,52]. The topology of VD was prepared using the GAFF with *Sobtop version 1.0 (dev3.1)* [53]. The RESP2 charges were added using *Multiwfn (dev3.8)* through a script [54]. The convergence status of the complex system was determined by calculating the free energy landscape. The lowest-energy frame of traces in the free energy trough was then extracted to predict protein–ligand interactions using the PLIP online server (https://plip-tool.biotec.tu-dresden.de/plip-web/plip/index/, accessed on 25 October 2022). The PyMOL was used to visualize the results from the PSE files provided by the PLIP website.

### 2.9. Calculations of MM-GBSA Binding Free Energy

The MM-GBSA tool was used to calculate the free energy between the protein and VD based on a single trajectory [55]. In this study, 1000 frames (corresponding to the last 10 ns of the trajectory) were employed for MM-GBSA calculations using the igb5 (GB-OBC2) model, with a salt concentration of 0.15M. In addition, the distance of residues between receptor and ligand for decomposition analysis was 4Å. The independent gradient model was subsequently applied using *Multiwfn (dev3.8)* to analyze non-covalent interactions, including hydrogen bonding, van der Waals forces, and π–π stacking [54].

## 3. Results

### 3.1. Inclusion of 23 RCTs Investigating the Effects of VD Supplementation in MetS

**Figure 2:**
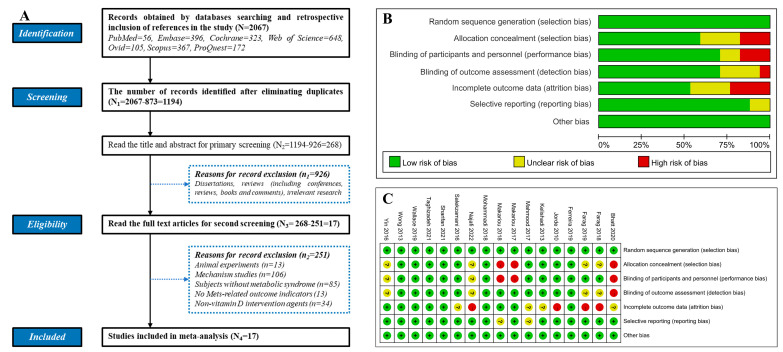
(**A**) Flow chart depicting the Preferred Reporting Items for Systematic Reviews and Meta-Analyses (PRISMA) study selection process. Assessment of bias in included studies through consensus between two raters. (**B**) Risk of bias graph: two raters’ judgments about each risk of bias item presented as percentages across all included studies. (**C**) Risk of bias summary: two raters’ judgments about each risk of bias item for each included study.

In our study, a total of 2067 relevant records were retrieved through online electronic databases. We then employed the EndNote X9 literature manager to exclude 873 duplicates. We further refined our selection process by excluding 926 irrelevant studies, which encompassed conferences, reviews, books, and comments, through the examination of titles and abstracts. After this initial screening, we identified 17 articles of interest after eliminating similar studies (Table S5) [16,56,57,58,59,60,61,62,63,64,65,66,67,68,69,70,71]. The details of our screening process are depicted in Figure 2A. Within the pool of seventeen studies, six of them incorporated two different doses of VD interventions, ultimately leading to the inclusion of 23 RCT groups [56,58,61,64,69,71]. Our comprehensive analysis also involved evaluating the risk of bias. The results of the Cochrane risk of bias assessment for the included studies are presented in the risk of bias graph (Figure 2B) and the risk of bias summary (Figure 2C). In total, the collective participation of 2165 subjects in these studies were observed, with follow-up loss rates ranging from 0% to 32.23%. For a more detailed overview of the primary characteristics of the included studies, please refer to Table S5.

### 3.2. Effects of VD Supplementation on Lipid Profiles

**Figure 3:**
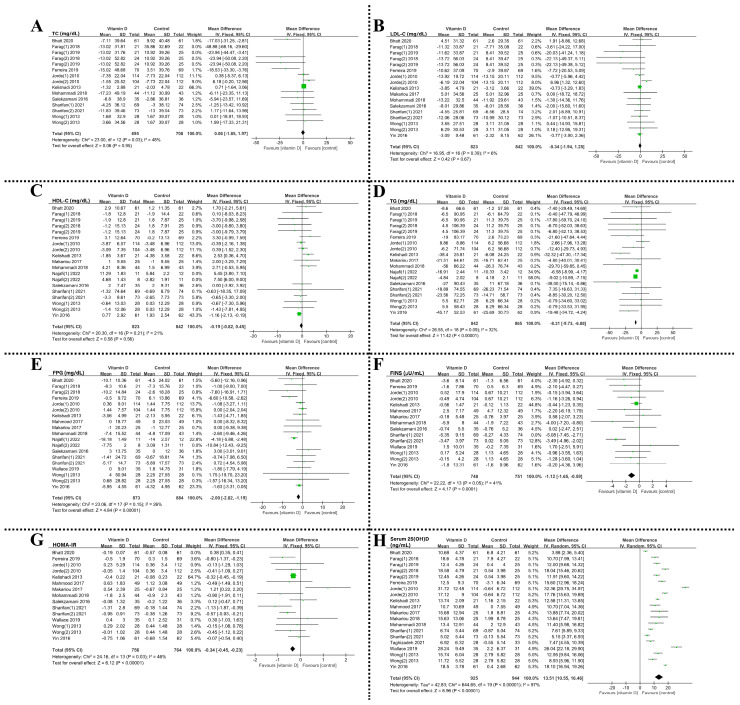
Forest plot for the effect of VD supplementation on outcome indicators of lipid profiles (**A**–**D**), insulin resistance (**E**–**G**), and serum 25(OH)D levels (**H**). Abbreviations: TC, total cholesterol; LDL-C, low-density lipoprotein cholesterol; HDL-C, high-density lipoprotein cholesterol; TG, triglyceride; FPG, fasting plasma glucose; FINS, fasting insulin; HOMA-IR, homeostasis model assessment of insulin resistance. Note: Squares and diamonds are typically used to represent effect sizes for each study and for all studies. In addition, the width of the squares indicates the weights for each study.

*Total cholesterol, TC.* Among the 15 RCTs analyzed in Figure 3A, a high degree of heterogeneity was observed (I^2^ = 73%, *p* < 0.00001). Sensitivity analysis revealed that the studies by Farag (1) et al. (2018) and Farag (1) et al. (2019) were the primary sources of heterogeneity due to the inclusion of additional outdoor physical activity alongside a daily 2000 IU VD supplement [61,64]. After excluding these two studies, the remaining trials showed moderate heterogeneity (I^2^ = 48%, *p* = 0.03) based on a fixed-effects model. The results indicated a non-significant increase in TC with VD intervention compared to control groups (MD = 0.06, 95% CI (−1.85, 1.97), *p* = 0.95) in mg/dL.

*Low-density lipoprotein cholesterol, LDL-C*. The test for heterogeneity (I^2^ = 6%, *p* = 0.39) of the 17 RCTs (Figure 3B) suggested that there was no heterogeneity among studies. The result showed that the VD intervention reduced LDL-C compared to the control groups but was not statistically significant (MD = −0.34, 95% CI (−1.94, 1.25), *p* = 0.67) in mg/dL.

*High-density lipoprotein cholesterol, HDL-C.* The analysis of 19 RCTs in Figure 3C revealed high heterogeneity (I^2^ = 86%, *p* < 0.00001) among the studies. After conducting a sensitivity analysis and excluding Najafi (1) et al. (2022) and Najafi (2) et al. (2022) [56], which involved weekly VD supplementation of 50,000 IU, with Najafi (1) et al. (2022) also including additional aerobic training for 8 weeks, a fixed-effects model (I^2^ = 21%, *p* = 0.21) was used to combine effect sizes. The observed heterogeneity in combined HDL-C effect sizes may be due to the high-dose VD supplementation. The results indicated that VD intervention reduced HDL-C levels compared to the control groups, but this reduction was not statistically significant (MD = −0.19, 95% CI (−0.82, 0.45), *p* = 0.56) in mg/dL.

*Triglyceride, TG.* The test for heterogeneity (I^2^ = 32%, *p* = 0.09) of the 19 RCTs (Figure 3D) indicated a modest degree of heterogeneity among the included studies. The results showed that VD intervention significantly reduced TG compared to control groups (MD = −8.31, 95% CI (−9.73, −6.88), *p* < 0.00001) in mg/dL.

### 3.3. Effects of VD Supplementation on Insulin Resistance

***Fasting plasma glucose, FPG.*** The test for heterogeneity (I^2^ = 85%, *p* < 0.00001) of the 19 RCTs (Figure 3E) indicated a high degree of heterogeneity among the included studies. Effect sizes were combined using a fixed model (I^2^ = 26%, *p* = 0.15) after Najafi (2) et al. (2022) [56] was removed by sensitivity analysis. The results showed that VD intervention significantly reduced FPG levels in subjects compared to control groups (MD = −2.00, 95% CI (−2.82, −1.19), *p* < 0.00001) in mg/dL.

***Fasting insulin, FINS***. The test for heterogeneity (I^2^ = 57%, *p* = 0.003) of the 15 RCTs (Figure 3F) indicated a moderate degree of heterogeneity among the studies. After removing Sharifan (2) et al. (2021) [58] by sensitivity analysis, we used a fixed model (I^2^ = 43%, *p* = 0.04) to combine effect sizes. The results showed that VD intervention significantly reduced FINS levels in subjects compared to control groups (MD = −1.00, 95% CI (−1.55, −0.45), *p* = 0.0003) in µU/mL.

***Homeostasis model assessment of insulin resistance, HOMA-IR***. The test for heterogeneity (I^2^ = 92%, *p* < 0.00001) of the 15 RCTs (Figure 3G) indicated a high degree of heterogeneity among the studies. After removing Bhatt et al. (2020) [59] through sensitivity analysis, we used a fixed model (I^2^ = 46%, *p* = 0.03) to combine effect sizes. In the study by Bhatt et al. (2020) [59], participants received 60,000 IU of VD supplementation per week for 8 weeks, along with daily calcium carbonate supplementation (1 mg/day). The high-dose VD supplementation and additional calcium carbonate intake may be the leading cause of heterogeneity. The results indicated a significant improvement in glucose homeostasis with VD supplementation compared to control groups (MD = −0.34, 95% CI (−0.45, −0.23), *p* < 0.00001).

### 3.4. VD Supplementation Effects on Serum 25(OH)D, Obesity Biomarkers, and Blood Pressure

The study included serum 25(OH)D (Figure 3H), blood pressure (Figure S1A,B), and obesity (Figure S1C,D) as secondary outcome indicators due to the controversy surrounding the effect of VD supplementation on biomarkers related to obesity [72]. The detailed descriptions of these results can be found in the Supplementary Materials Section S2.2 and Section S2.3.

### 3.5. Lipid Profile Subgroup Analysis in MetS Based on Serum 25(OH)D Levels

The current primary international clinical diagnostic criteria for determining serum 25(OH)D status are the ***Endocrine Society Clinical Practice Guidelines*** [73]. Recently, Ashley et al. [74] have stated that the ***Global Consensus*** (Table S6) recommends classifying serum 25(OH)D sufficiency as levels above 20 ng/mL, serum 25(OH)D insufficiency as levels between 12 and 20 ng/mL, and serum 25(OH)D deficiency as levels below 12 ng/mL. Due to the heterogeneity in the effects of VD supplementation on lipids, the lipid profiles (TC, LDL-C, HDL-C, TG) of subjects were categorized into three subgroups (Table 1) for analysis, based on their baseline serum 25(OH)D levels. When subjects’ serum 25(OH)D levels were below 12 ng/mL, VD supplementation significantly reduced their TC (MD = −32.34, 95% CI (−43.52, −21.15), *p* < 0.00001 *) and LDL-C levels (MD = −15.49, 95% CI (−27.22, −3.77), *p* = 0.01 *), indicating that VD supplementation can significantly improve lipid profiles in individuals with severe vitamin D deficiency.

In a subgroup analysis of serum 25(OH)D levels ranging from 12 to 20 ng/mL, VD supplementation resulted in a significant reduction in TG levels (MD = −8.43, 95% CI (−9.88, −6.98), *p* < 0.00001 *), along with an increase in HDL-C levels (MD = 1.88, 95% CI (1.24, 2.52), *p* < 0.00001 *) among the subjects. However, the subgroup analysis revealed a high heterogeneity of HDL-C (I^2^ = 91%, *p* < 0.00001) among subjects with baseline serum 25(OH)D levels of 12~20 ng/mL. The source of this heterogeneity was identified as Najafi et al. (2022) [56], as confirmed through a sequential exclusion of individual studies. The intervention in this study involved aerobic training with high-dose VD supplementation (50,000 IU/week for 8 weeks). In another study, Kashkooli et al. (2019) [75] found through subgroup analysis that combined calcium and VD supplementation (400 IU/day of VD and 600 mg/day of calcium for 8 weeks) exhibited significant efficacy in improving HDL-C in overweight or obese subjects. However, due to the variations in the interventions studied, the results should be interpreted with caution.

### 3.6. Candidate and Functional Targets of VD Anti-MetS

**Figure 4:**
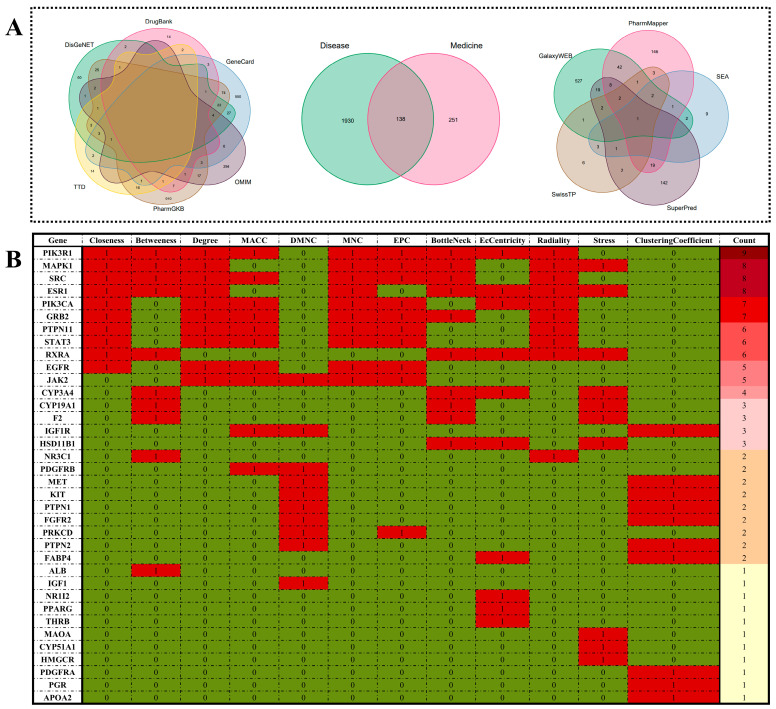
(**A**) Venn diagram of VD against MetS. Disease-related targets: DrugBank (32), TTD (45), OMIM (330), PharmGKB (1093), Genecard (697), DisGeNET (153). Medicine-related targets: GalaxySagittarius-AF (83), Super-PRED (186), PharmMapper (287), Swiss Target Prediction (31), SEA (26). (**B**) Hub targets of VD against MetS by cytoHubba according to 12 different algorithms. Note: Red color indicates core genes screened by the algorithm, while green color indicates non-core genes.

In our study, we initially screened potential disease-related targets from six databases: Genecards, DisGeNET, TTD, OMIM, PharmGkb, and DrugBank. This yielded different numbers of potential targets from each database: 697, 153, 45, 330, 1093, and 32, respectively. After merging the targets from these six disease databases, we removed any duplicate entries, resulting in a total of 2068 targets related to MetS. Furthermore, we employed five online databases (GalaxySagittarius-AF, Super-PRED, PharmMapper, Swiss Target Prediction, and SEA) to predict potential therapeutic targets for VD. These databases provided varying numbers of potential targets: 83, 186, 287, 31, and 26, respectively. In the final compilation, a total of 389 predicted targets were aggregated from these five databases, with careful removal of any duplicate entries. As depicted in Figure 4A, 138 mapped targets are displayed in a Venn diagram. The proteins at the intersection were then imported into *Cytoscape (version dev-3.9.1)* to evaluate the topological parameters associated with the interaction of VD with MetS. The heat map in Figure 4B illustrates our screening of 36 hub targets using 12 topological parameters.

### 3.7. Identifying 14 Core Targets through GO Annotation and KEGG Enrichment Analysis

**Figure 5:**
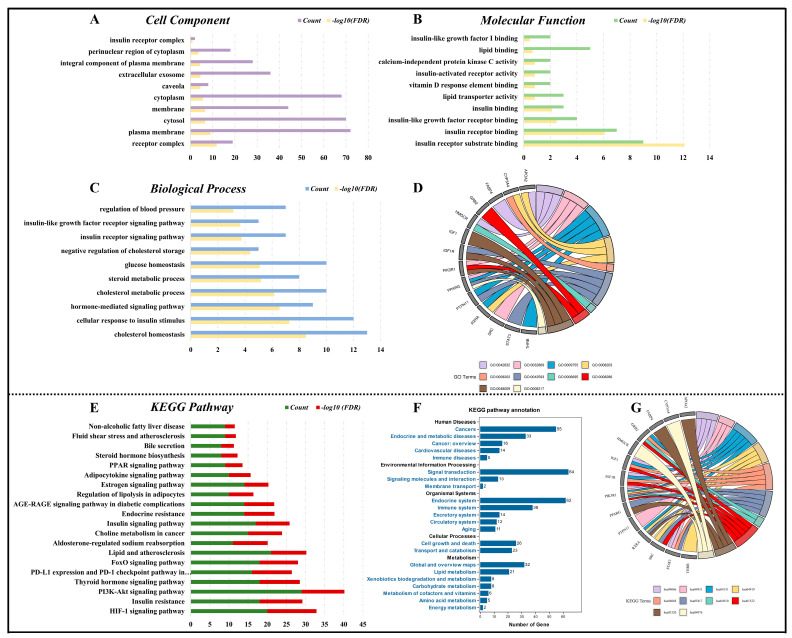
Key cell components, molecular functions, and biological processes of VD against MetS illustrated in bar diagrams (**A**–**C**). Identification of 14 core genes involved in MetS-related biological processes. (**D**). Relevant signaling pathway annotations of VD against MetS characterized in bar diagram (**E**,**F**). Exploration of the 14 core genes involved in KEGG signaling pathways associated with MetS (**G**).

In our GO functional enrichment analysis, we observed the following results: 55 entries enriched for cell components, 108 entries enriched for molecular functions, and 388 entries enriched for biological processes. Our findings revealed that the primary cell components of core targets included the plasma membrane, cytosol, and insulin receptor complex (Figure 5A). Regarding the molecular functions of the core targets, they were associated with lipid transporter activity, VD response element binding, and insulin binding (Figure 5B). In terms of biological processes, the core targets played roles in regulating cholesterol homeostasis, glucose homeostasis, and responding to insulin stimuli (Figure 5C).

In addition, we identified 20 KEGG pathways (Figure 5E) associated with core targets. By analyzing the primary signaling pathways and KEGG pathway annotations (Figure 5F), we uncovered 14 core regulatory genes linked to MetS. These 14 core genes were involved in various biological processes and contributed to the KEGG pathway associated with the anti-MetS mechanisms of VD, as depicted in the circle diagram (Figure 5D,G).

### 3.8. Screening of Three Key Targets Involved in Lipid Metabolism

By searching for annotation information on the 14 core genes in the NCBI database, we identified three genes primarily associated with MetS and heavily involved in lipid metabolism. These three genes were peroxisome proliferator-activated receptor gamma (PPARγ, PDB: 6FZG) [76], fatty acid binding protein 4 (FABP4, PDB: 5EDB) [77], and 3-hydroxy-3-methylglutaryl-CoA reductase (HMGCR, PDB: 1HW8) [78]. They may modulate the PPAR signaling pathway and inhibit bile secretion. Subsequently, we retrieved their corresponding target proteins from the PDB database. To validate the reliability of our docking method, we employed the LibDock module of *Discovery Studio 2019* to redock the proto-ligands to the three target proteins. As shown in Table 2, the RMSDs of backbone stacking obtained from redocking the three proteins were less than 2 Å, indicating that the docking method had good reliability. The initial conformation of molecular docking was then used in dynamics simulations to investigate the non-covalent interactions between the protein and the ligand at the microscopic level.

### 3.9. Exploring Stable Conformations of Six Complex Systems through Free Energy Landscapes

As depicted in Figure S4, the RMSD of the six systems ranged from 0.1 to 0.3 nm, indicating that they essentially reached a stable state within the final 20 ns. During the last 20 ns of the MD simulation, the radius of gyration (Rg) of the 1HW8–VD and 5EDB–VD complexes was smaller than that of the original apo proteins, indicating stable binding of VD to the proteins. Conversely, the Rg of 6FZG–apo increased, possibly due to the high flexibility of the loop region in the 6FZG-apo tail. However, the 6FZG–VD complex remained stable within the range of 2 nm during the simulation, indicating tight binding of VD to 6FZG and a reduction in the flexibility of its tail loop. We calculated the root-mean-square fluctuation (RMSF) of each residue to assess the impact of VD binding on protein structural flexibility. In Figure S4, the RMSF of the six complexes and their corresponding apo-proteins ranges from 0 to 0.8 nm, with a consistent fluctuation trend. This indicated that the protein conformations tended to stabilize after binding VD. However, the residues of 6FZG–apo protein displayed notable flexibility in the loop region (Lys-293–Lys-303). Importantly, upon binding with VD, the RMSF of 6FZG–VD decreased significantly, indicating the formation of a stable complex and reduced flexibility fluctuation. This result aligned with the Rg findings mentioned earlier.

To investigate the convergence of protein free energy in MD simulations, we plotted the co-correlation matrix plot and free energy landscape (FEL) using the RMSD and gyration of protein backbones as horizontal and vertical coordinates (Figures S5 and S6). In the FEL, the size and shape of the free energy trough (blue region) revealed the stability of the protein conformation during the MD simulation. The smaller and more concentrated blue regions indicate a more stable protein conformation. In Figure S6, the Gibbs free energy values of the stable conformations ranged from 0 to 14 kJ/mol, and each system exhibited a distinct free energy valley, indicating that all complex systems had reached a stable state. Then, the frame with the lowest energy from each free energy trough was extracted as the representative conformation to analyze protein–ligand interactions.

### 3.10. Protein–Ligand Interactions of Six Complex Systems

**Figure 6:**
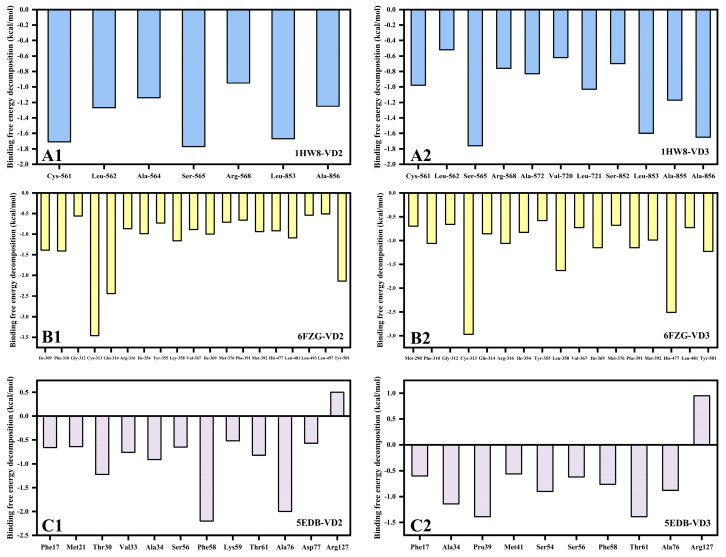
The key residues of contribution binding energy of HMGCR (**A1**,**A2**), PPARγ (**B1**,**B2**), and FABP4 (**C1**,**C2**). The non-contributing residues were removed (per-residue threshold: 0.50 kcal/mol).

According to the lowest energy conformation of the FEL, it was known that Arg-568 of the 1HW8 protein formed a hydrogen bond with the hydroxyl group of VD_2_ (Figure S7A1). Additionally, the Arg-568 of the 1HW8 protein formed an alkyl bond with VD_3_ (Figure S7A2). The Leu-853 and Ala-856 of the 1HW8 protein formed alkyl bonds with both VD_2_ and VD_3_ (Figure S7A). Furthermore, the absolute value of the binding free energy contribution of residues Arg-568, Leu-853, and Ala-856 in the binding free energy decomposition was greater than 1 kcal/mol (Figure 6), suggesting that these residues played a crucial role in 1HW8–VD interactions.

The Gln-314 of the 6FZG protein formed a conventional hydrogen bond with VD_2_ (Figure S7B1). The His-477 and Tyr-501 of the 6FZG protein formed conventional hydrogen bonds with VD_3_ (Figure S7B2). The Cys-313 of the 6FZG protein formed an alkyl bond with both VD_2_ and VD_3_ (Figure S7B). In the binding free energy decomposition, 0.5 kcal/mol was used as the threshold for delineating the residue free energy contribution. As depicted in Figure 6, the 6FZG–VD complex had the largest number of residues for free energy decomposition, with Cys-313 and Tyr-501 contributing more than 1 kcal/mol each to the binding free energy.

The Phe-58 of the 5EDB protein formed a pi–alkyl bond with VD_2_ (Figure S7C1). The Pro-39 of the 5EDB protein formed an alkyl bond with VD_3_ (Figure S7C2). Additionally, both Ala-34 and Ala-76 within the A chain of the 5EDB protein formed alkyl bonds with both VD_2_ and VD_3_ (Figure S7C). In summary, our results indicated that the stability of the formed complexes followed the order: 6FZG–VD > 5EDB–VD > 1HW8–VD, revealing that the binding of VD with 6FZG was more favorable than with 5EDB or 1HW8 (Figure S8).

### 3.11. Van der Waals Forces Drive Protein–Ligand Interactions in Six Complexes

**Figure 7:**
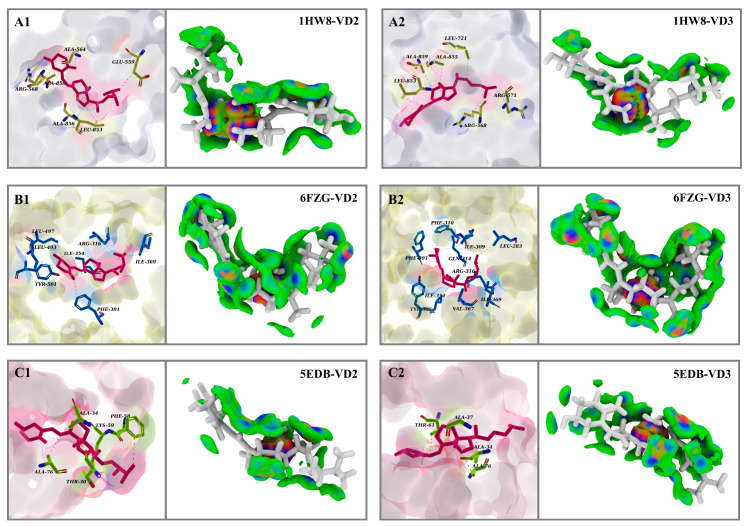
The lowest energy binding conformation and δ_g_^inter^ iso-surface analysis of VD with 1HW8 (**A1**,**A2**), 6FZG (**B1**,**B2**), and 5EDB (**C1**,**C2**) in the identified core targets of HMGCR, PPARγ, and FABP4 in MetS.

In Figure S9, the scatter plot illustrates the relationship between δg_inter and δg_intra concerning the sign(λ2)ρ for the independent gradient model (IGM). The black scatter points (δg_intra) represent intra-molecular interactions, while the red scatter points (δg_inter) represent inter-molecular interactions. The distributions of these scatter points characterize the intra-molecular covalent bonding, repulsive interactions, and inter-molecular hydrogen bonding interactions within the system. To visualize these interactions, we rendered the iso-surfaces for the IGM. The blue, green, and red iso-surfaces represent hydrogen bonding, van der Waals, and repulsive interactions, respectively. In Figure 7, the iso-surfaces of the six complex systems are predominantly green, suggesting that van der Waals forces are the primary forces responsible for maintaining protein–ligand binding.

In Table 3, the binding free energy Δ*G_bind_* is composed of Δ*E_vdW_*, Δ*E_ele_*, Δ*G_pol_*, and Δ*G_non-pol_*. Among the six complex systems, polar solvation free energy (Δ*G_pol_*) had an unfavorable contribution to ligand–protein binding. In contrast, van der Waals interactions (Δ*E_vdW_*), electrostatic interactions (Δ*E_ele_*), and the non-polar solvation free energy (Δ*G_non-pol_*) provided favorable contributions to ligand–protein binding, with Δ*E_vdW_* contributing the most to the binding free energy. These findings were consistent with the results of the IGM. Therefore, it could be speculated that van der Waals forces were the driving forces behind the interactions between VD and the core protein receptors.

## 4. Discussion

MetS refers to a pathological state of abnormal metabolism, including central obesity, glucose intolerance, and abnormal lipid metabolism. Due to the diversification of diets and the modernization of lifestyles, the incidence of MetS is not only increasing globally but also affecting individuals at a younger age. As a dietary supplement and nutritional fortifier, VD improves the nutritional value of foods and helps consumers more easily meet their daily VD requirements. VD plays a crucial role in regulating physiological processes, but its relationship with MetS biomarkers remains controversial due to a lack of definitive scientific evidence. This enlightens us to explore new methodologies for investigating VD as a potential treatment for MetS.

In this study, we employed meta-analysis, a commonly used method in dietary nutrition research, to examine the effects of VD supplementation on lipid profiles and insulin resistance in individuals with MetS. Our results indicated that VD supplementation effectively reduced serum TG concentrations during the early intervention phase. However, we did not observe statistically significant changes in other vital biomarkers for monitoring lipid metabolism, such as serum LDL-C, HDL-C, and TC levels. These findings were consistent with AlAnouti, F. et al. [79]. Furthermore, our study revealed that VD supplementation significantly reduced FPG levels, improved insulin resistance, and lowered systolic blood pressure. As a result, we hypothesize that VD may interact with multiple targets in vivo, influencing various metabolic and disease-related pathways. To explore these mechanisms further, we utilized in silico methods, including GO and KEGG pathway enrichment analysis, which indicated that VD may primarily modulate bile secretion and the PPAR signaling pathway. Additionally, through molecular docking and dynamics simulation, we identified HMGCR, FABP4, and PPARγ as primary targets.

HMG-CoA reductase, a rate-limiting enzyme in cholesterol synthesis, was found to interact with VD through hydrogen and alkyl bonds. Previous studies have also demonstrated that VD directly influences the lipid profile by inhibiting the enzymatic activity of HMGCR [13,80]. FABP4, involved in fatty acid uptake and metabolism, was shown to form alkyl bonds with VD. This interaction implies that VD may act as an inhibitor of FABP4, potentially improving lipid metabolism in MetS. PPARγ, a regulator of adipocyte differentiation, exhibited conventional hydrogen bonding with VD. The residue Cys-313 of PPARγ contributed significantly to the binding free energy decomposition. The interactions between VD and PPARγ may play a role in maintaining metabolic homeostasis.

Recent research has linked reduced serum VD levels to an increased risk of MetS and cardiovascular events [81,82,83,84]. Serum 25(OH)D deficiency is associated with dyslipidemia, inflammation, glucose and fatty acid metabolism disruption, insulin resistance, and chronic metabolic disorders [85]. As a result, dietary VD supplementation provides beneficial health effects for individuals with MetS.

In brief, our study represents a pioneering effort to combine meta-analysis and in silico approaches to comprehensively investigate the pharmacological mechanisms and potential targets of VD in individuals with MetS, focusing on dyslipidemia and insulin resistance.

## 5. Conclusions

We demonstrate the beneficial effects of VD supplementation on MetS, including the regulation of lipid profiles and attenuation of insulin resistance. Given the intricate nature of the occurrence and progression of MetS, there has been increased interest in employing multitarget and multichannel therapeutic strategies, such as dietary VD supplementation, for the prevention of MetS. Through in silico approaches, we predict three key targets (HMGCR, FABP4, PPARγ), revealing that VD exerts a therapeutic influence on MetS by inhibiting bile secretion and modulating the PPAR signaling pathway. Our findings provide valuable insights into the potential use of VD as a dietary supplement for preventing MetS. In summary, our research on the role of VD in addressing MetS within the field of nutrition can enhance subjects’ quality of life and raise public awareness of health. Future research can involve larger, high-quality RCTs to further elucidate the effects of VD in chronic clinical conditions or other diseases.

## Figures and Tables

**Table 1:** Subgroup characteristics of VD supplementation on lipid profiles.

Baseline serum 25(OH)D (ng/mL)	Number	WMD (95% CI)	*p* within Group	*p* Heterogeneity	I^2^
Subgroup analyses of VD supplementation on TC level (mg/dL)					
<12	187	−32.34 (−43.52, −21.15)	<0.00001 *	0.23	30%
12~20	736	−0.13 (−2.31, 2.05)	0.91	0.16	34%
>20	529	2.45 (−1.69, 6.60)	0.25	0.25	27%
Subgroup analyses of VD supplementation on LDL-C level (mg/dL)					
<12	187	−15.49 (−27.22, −3.77)	0.01 *	**0.59**	**0%**
12~20	909	−0.68 (−2.49, 1.12)	0.46	**0.99**	**0%**
>20	529	2.46 (−1.21, 6.12)	0.19	0.12	53%
Subgroup analyses of VD supplementation on HDL-C level (mg/dL)					
<12	187	−2.65 (−6.11, 0.80)	0.13	**0.90**	**0%**
12~20	951	1.88 (1.24, 2.52)	<0.00001 *	<0.00001 *	91%
>20	529	0.35 (−0.86, 1.55)	0.57	0.26	26%
Subgroup analyses of VD supplementation on TG level (mg/dL)					
<12	187	−8.49 (−30.92, 13.93)	0.46	**0.96**	**0%**
12~20	951	−8.43 (−9.88, −6.98)	<0.00001 *	0.05 *	45%
>20	529	−3.79 (−12.46, 4.88)	0.39	0.07	62%

The chi-square (χ^2^) heterogeneity test was used to test whether there were significant differences in effect sizes between studies. A * means a significant difference (*p* < 0.05). Generally, I^2^ values below 25% indicate low heterogeneity, between 25% and 50% indicate moderate heterogeneity, and above 50% indicate high heterogeneity. Boldface indicates that there is no heterogeneity between studies. Abbreviations: CI, confidence interval; TC, total cholesterol; LDL-C, low-density lipoprotein cholesterol; HDL-C, high-density lipoprotein cholesterol; TG, triglyceride; WMD, weighted mean difference.

**Table 2:** The result of molecular docking through *Discovery Studio 2019*.

Core Gene	PDB	Resolution (Å)	Redocking (Å)	LibDock Score	
				VD_2_	VD_3_
PPARγ	6FZG	2.10	OL: EDK *, RMSD = 0.7616	127.72	119.91
FABP4	5EDB	1.18	OL: 5M8 *, RMSD = 0.7477	102.58	98.57
HMGCR	1HW8	2.10	OL: 114 *, RMSD = 1.2337	101.60	108.28

OL: original ligand; RMSD: root-mean-square deviation. The symbol * indicates that the original ligand has been experimentally verified.

**Table 3:** The binding free energies of VD to proteins calculated through MM-GBSA.

Energy ^a^	1HW8–VD2	1HW8–VD3	6FZG–VD2	6FZG–VD3	5EDB–VD2	5EDB–VD3
Δ*E_vdW_*	−40.17 ± 2.30	−43.72 ± 2.85	−57.37 ± 2.70	−58.74 ± 2.67	−40.78 ± 2.81	−48.48 ± 2.10
Δ*E_ele_*	−11.84 ± 4.41	−4.47 ± 4.38	−13.81 ± 3.14	−14.83 ± 2.62	−2.36 ± 1.55	−6.22 ± 2.67
Δ*G_pol_*	33.35 ± 3.24	26.10 ± 3.69	25.69 ± 1.17	26.92 ± 1.4	21.75 ± 2.04	31.13 ± 2.04
Δ*G_non-pol_*	−4.96 ± 0.22	−5.55 ± 0.31	−7.81 ± 0.22	−7.79 ± 0.21	−5.18 ± 0.39	−6.18 ± 0.22
Δ*G_bind_*	−23.63 ± 2.96	−27.63 ± 3.42	−53.30 ± 3.31	−54.44 ± 3.09	−26.56 ± 2.24	−29.75 ± 2.47

^a^ All units are reported in kcal/mol (mean ± SD). Δ*G_pol_* and Δ*G_non-pol_* mean polar and non-polar contributions to the solvation free energy, separately. Δ*E_vdW_* and Δ*E_ele_* represent the van der Waals and electrostatic contributions, respectively. Δ*G_bind_* refers to the binding free energy ${(∆G}_{bind}={∆E}_{vdW}+{∆E}_{ele}+{∆G}_{pol}+{∆G}_{non-pol})$.

## Data Availability

Data are contained within the manuscript or Supplementary Materials.

## Supplementary Materials

The following supporting information can be downloaded at: https://www.mdpi.com/article/10.3390/foods12213973/s1, Table S1 The search strategy of PubMed; Table S2 The Cochrane risk bias assessment tool; Table S3 Unit conversions for relevant outcome indicators; Table S4 Target prediction of VD for the treatment of MetS and online url; Table S5 The basic characteristics of the included studies; Table S6 Diagnostic criteria for serum 25(OH)D; Table S7 The result of molecular docking through AutoDock 4.2; Figure S1 Forest plot for fixed-effects model of the effect of VD supplementation on outcome indicators for blood pressure (A–B) and obesity (C–D); Figure S2 The Egger regression test for bias in 20 RCTs on serum 25(OH)D levels; Figure S3 Funnel plot of VD supplementation on lipids profile (A), insulin resistance (B), blood pressure (C) and anthropometry-related (D) outcome indicators in subjects; Figure S4 The results of molecular dynamics simulations. RMSD (A1, B1, C1), Rg (A2, B2, C2), RMSF (A3, B3, C3) of three proteins in free and combined states with VD. The simulation time was 100 ns for all systems except for the complex 1HW8–VD system, which was 200 ns; Figure S5 Plot of the co-correlation matrix with principal component analysis for the six complex systems; Figure S6 Free energy landscape of six protein–ligand complex systems; Figure S7 Predicting protein–ligand 2D interactions diagrams through Discovery Studio 2019; Figure. S8 Calculation of binding free energies for six complex systems using the MM-GBSA method; Figure S9 Visual scatter maps of δ_g_ ^inter^ and δ_g_ ^intra^ vs. sign(λ_2_)*p* in independent gradient model. The method of supplementary materials has been mentioned at [86].

## Abbreviations

HMGCR	3-Hydroxy-3-methylglutaryl-CoA reductase
FABP4	Fatty acid binding protein 4
PPARγ	Peroxisome proliferator-activated receptor gamma
ROR α/γ	Retinoic acid receptor-related orphan receptor alpha/gamma
LXR α/β	Liver X receptor alpha/beta
VDR	Vitamin D receptor
MeSH	Medical subject headings
VD	Vitamin D
NCEP-ATP Ⅲ	National Cholesterol Education Program Adult Treatment Panel III
IDF	International Diabetes Federation
MetS	Metabolic syndrome
CVD	Cardiovascular disease
RCTs	Randomized controlled trials
MD	Mean difference or molecular dynamics (simulation)
SD	Standard deviation
FDR	False discovery rate
TC	Total cholesterol
LDL-C	Low-density lipoprotein cholesterol
HDL-C	High-density lipoprotein cholesterol
TG	Triglyceride
FPG	Fasting plasma glucose
FINS	Fasting insulin
HOMA-IR	Homeostasis model assessment of insulin resistance
WC	Waist circumference
BMI	Body mass index
KEGG	Kyoto Encyclopedia of Genes and Genomes
GO	Gene Ontology
MM-GBSA	Molecular mechanics generalized Born surface area
FEL	Free energy landscape
IGM	Independent gradient model
RMSD	Root-mean-square deviation
RMSF	Root-mean-square fluctuation
Rg	Radius of gyration
