# Supplementary material for: Insight into the Interaction Mechanism of Vitamin D against Metabolic Syndrome: A Meta-Analysis and In Silico Study
Source: Foods. 2023 Oct 30;12(21):3973. doi: 10.3390/foods12213973 (PMC10649035; doi:10.3390/foods12213973)
Supplement: Supplementary file 1 [file foods-12-03973-s001.zip › Supplementary Materials.docx]

Supplementary Materials

Insight into the Interaction Mechanism of Vitamin D against Metabolic Syndrome: A Meta-Analysis and In Silico Study

Yuting Xia ^1,†^, Yuandong Yu ^1,†^, Yi Zhao ^1^, Zhifen Deng ^1^, Lei Zhang ^2,^* and Guizhao Liang ^1,^*

^1^ Key Laboratory of Biorheological Science and Technology, Ministry of Education, Bioengineering College, Chongqing University, Chongqing 400044, China; 202219021088t@stu.cqu.edu.cn (Y.X.); yuandongncm@163.com (Y.Y.); 20221901021@stu.cqu.edu.cn (Y.Z.); 202119021155t@cqu.edu.cn (Z.D.)

^2^ College of Life Science, Chongqing Normal University, Chongqing 401331, China

* Correspondence: leizhang0215@126.com (L.Z.); gzliang@cqu.edu.cn (G.L.);
Tel.: +86-(023)-65916239 (L.Z.); +86-(023)-65102507 (G.L.)

# 1. Methods

This study was carried out according to the Preferred Reporting Items for Systematic Reviews and Meta-Analyses (PRISMA) statement and following a predefined protocol that was registered with PROSPERO (registration number CRD42022322365) [1].

## **1.1 Criteria for inclusion and exclusion**

***Criteria for included studies were as follows:***

(1) The study subjects were diagnosed with MetS according to National Cholesterol Education Program Adult Treatment Panel III (NCEP-ATP Ⅲ) or International Diabetes Federation (IDF). In addition, pre-diabetic and obese populations were included in the study.

(2) The experimental group intervention was any form of VD_3_ or VD_2_, and the control group intervention did not contain any VD supplementation.

(3) The outcome indicators included the biomarkers of lipid profile (TC, LDL-C, HDL-C, or TG) and insulin resistance.

(4) Only RCTs lasting at least 4 weeks were included to ensure that the intervention had sufficient time to have an effect.

***Criteria for excluded studies were as follows:***

(1) Repeated reports of the same study.

(2) Invited reviews, academic conferences, dissertations and review papers on studying the mechanisms of VD metabolism.

(3) The subjects of the study were animals.

(4) The subjects had a variety of diseases such as non-alcoholic fatty liver, chronic kidney dialysis patients, type I diabetes and osteoporosis, among other pathologies that were not relevant to the study.

## **1.2. Search strategy**

**Table S1.** The search strategy of PubMed.

| #1 | "Metabolic Syndrome"[Mesh] |
| --- | --- |
| #2 | "Metabolic Syndrome*"[Title/Abstract] |
| #3 | #1 OR #2 |
| #4 | "Vitamin D"[Mesh] |
| #5 | "Cholecalciferol*"[Title/Abstract] |
| #6 | "Ergocalciferol*"[Title/Abstract] |
| #7 | #4 OR #5 OR #6 |
| #8 | "Lipids"[Mesh] |
| #9 | "Cholesterol"[Mesh] |
| #10 | "High-density lipoprotein"[Text Word] |
| #11 | "Low-density lipoprotein"[Text Word] |
| #12 | #8 OR #9 OR #10 OR #11 |
| #13 | "Randomized controlled trial"[Publication Type] |
| #14 | "Randomized Controlled Trials as Topic"[MeSH] |
| #15 | #13 OR #14 |
| #16 | #3 AND #7 AND #12 AND #15 |

## 1.3. Bias assessment

The risk of bias (Table S2) was evaluated according to the Cochrane Handbook (version 5.1.0).

**Table S2.** The Cochrane risk bias assessment tool.

| **Risk of bias** | |
| --- | --- |
| Random sequence generation | Whether the method of generating the random assignment sequence is described in detail so that comparability between groups can be assessed |
| Allocation concealment | Whether the personnel implementing the assignment strictly enforce the results of random numbers |
| Blinding of participants and personnel  Blinding of outcome assessment | Whether outcome assessment is blinded (subject, investigator, assessor) |
| Incomplete outcome data | Whether dropout cases (missed visits, withdrawals or non-compliance) are kept to a very small range to ensure a balanced number of dropout cases between groups |
| Selective reporting | Selective reporting of positive findings and concealment of adverse grey findings for publication |
| Other bias | Conflict of interest, small sample size, unbalanced baseline |

## 1.4. Statistical analysis

Check the unit conversions of relevant outcome indicators to unify units:

**Table S3.** Unit conversions for relevant outcome indicators.

| **Outcome indicators** | **Unify units** |
| --- | --- |
| Serum 25(OH)D | 1 nmol/L= 0.4 ng/mL |
| TC, LDL-C, HDL-C | 1 mmol/L = 38.67 mg/dL |
| TG | 1 mmol/L = 88.57 mg/dL |
| Fasting blood glucose | 1 mmol/L = 18.02 mg/dL |
| Fasting insulin | 1 pmol/L = 0.153 µU/mL |

## 1.5 Prediction of VD anti-MetS targets

We used the following databases to predict relevant targets:

**Table S4.** Target prediction of VD for the treatment of MetS and online url.

| The MetS-driven targets acquired from the disease databases | |
| --- | --- |
| DisGeNET [2] | http://www.disgenet.org/ |
| GeneCards [3] | https://www.genecards.org/ |
| Online Mendelian Inheritance in Man (OMIM) [4] | http://www.omim.org/ |
| DrugBank [5] | https://www.drugbank.ca/ |
| Pharmacogenetics Knowledge Base (PharmGkb) [6] | https://www.pharmgkb.org/ |
| Therapeutic Target Database (TTD) [7] | http://bidd.nus.edu.sg/group/cjttd/ |
| The predicted targets of VD | |
| PharmMapper [8] | http://www.lilab-ecust.cn/pharmmapper/ |
| Swiss Target Prediction [9] | http://www.swisstargetprediction.ch/ |
| Similarity ensemble approach (SEA) [10] | https://sea.bkslab.org/ |
| Super-Pred [11] | https://prediction.charite.de/index.php/ |
| GalaxySagittarius-AF [12] | https://galaxy.seoklab.org/ |

# 2. Results

## 2.1. Basic information

**Table S5.** The basic characteristics of the included studies.

| **Author & year** | **Sample** | **Drop-out** | **Research population** | **Definition of MetS** | **Intervention** | **Duration** | **Daily dose equivalent** |
| --- | --- | --- | --- | --- | --- | --- | --- |
| Jorde 2010 [13] | 438 | 24.66% | Overweight or obese subjects | None | 40,000 IU/week of VD_3_ + 500mg/day of calcium | 1 year | 5714.29 IU/day |
|  |  |  |  |  | 20,000 IU/week of VD_3_ + 500mg/day of calcium |  | 2857.14 IU/day |
| Kelishadi 2013 [14] | 50 | 14.00% | Obese children and adolescents | None | 300,000 IU/week of VD_3_ | 12 weeks | 42857.14 IU/day |
| Wong 2013 [15] | 90 | 6.67% | MetS | NCEP-ATP III | 20,000 IU/week of VD_2_ | 8 weeks | 5714.29 IU/day |
|  |  |  |  |  | 40,000 IU/week of VD_2_ |  |  |
| Yin 2016 [16] | 126 | 2.38% | MetS | NCEP-ATP III | 700 IU/day of VD_3_ | 12 months | 700 IU/day |
| Salekzamani 2016 [17] | 80 | 11.25% | MetS | IDF criteria | 50,000 IU/week of VD_3_ | 4 months | 7142.85 IU/day |
| Mahmood 2017 [18] | 115 | 14.78% | MetS | IDF criteria | VD_3_ (60,000 IU/week for 8 weeks and 60,000 IU/month for 4 months) | 6 months | 5285.71 IU/day |
| Makariou 2017 [19] | 50 | 0% | MetS | NCEP-ATP III | 2000 IU/day of VD | 3 months | 2000 IU/day |
| Makariou 2018 [20] | 50 | 0% | MetS | NCEP-ATP III | 2000 IU/day of VD | 3 months | 2000 IU/day |
| Farag 2018 [21] | 120 | 23.33% | MetS | IDF criteria | 2000 IU/day of VD | 3 months | 2000 IU/day |
|  |  |  |  |  | 2000 IU/day of VD + 30 min/day of endurance PA |  |  |
| Mohammadi 2018 [22] | 90 | 3.33% | MetS | NCEP-ATP III | 1000 mg/day of calcium,  1000 IU/day of VD_3_ | 10 weeks | 1000 IU/day |
| Farag 2019 [23] | 90 | 22.22% | MetS | IDF criteria | 2000 IU/day of VD | 12 weeks | 2000 IU/day |
|  |  |  |  |  | 2000 IU/day of VD + 30 min/day of endurance PA |  |  |
| Ferreira 2019 [24] | 160 | 6.88% | Women menopause for at least 1 year | None | 1000 IU/day of VD_3_ | 9 months | 1000 IU/day |
| Wallace 2019 [25] | 66 | 3.03% | Prediabetes | ADA criteria | 3000 IU/day of VD_3_ | 26 weeks | 3000 IU/day |
| Bhatt 2020 [26] | 121 | 32.23% | Females with prediabetes | None | 60,000 IU/week of VD_3_ for 8 weeks + calcium carbonate | 78 weeks | VD deficiency (8671.43 IU/day);  Normal 25(OH)D (200 IU/day) |
| Taghizadeh 2021 [27] | 153 | 9.15% | MetS | IDF criteria | 1500 IU/day of nano-encapsulated VD | 10 weeks | 1500 IU/day |
| Sharifan 2021 [28] | 306 | 5.56% | Adults with abdominal obesity | IDF criteria | 1500 IU/day nano-encapsulated VD_3_ | 10 weeks | 1500 IU/day |
| Najafi 2022 [29] | 60 | 23.33% | Postmenopausal women with MetS | NCEP-ATP III | 50,000 IU/week of VD + AT | 8 weeks | 7143 IU/day |

| **Author & year** | **Age (year)** | | **BMI (kg/m^2^)** | | **Serum 25(OH)D (ng/mL)** | | **FPG (mmol/L)** | |
| --- | --- | --- | --- | --- | --- | --- | --- | --- |
|  | **Intervention** | **Control** | **Intervention** | **Control** | **Intervention** | **Control** | **Intervention** | **Control** |
| Jorde 2010 | 46.3 (11.30) | 48.9 (11.00) | 34.8 (4.00) | 35.1 (3.80) | 23.48 (8.48) | 23.52 (8.4) | 5.27 (0.57) | 5.34 (0.54) |
|  | 47.3 (11.90) |  | 34.4 (3.80) |  | 22.68 (8.48) |  | 5.25 (0.55) |  |
| Kelishadi 2013 | 10~16 | | 28.08 (1.06) | 27.81 (1.04) | 18.27 (2.04) | 17.91 (2.27) | 5.23 (0.30) | 5.12 (0.35) |
| Wong 2013 | 62.29 (10.63) | 65.07 (11.31) | 26.90 (3.65) | 25.88 (3.40) | 14.29 (3.35) | 16.20 (2.99) | 6.82 (2.96) | 6.32 (1.48) |
|  | 63.61 (13.25) |  | 27.63 (4.20) |  | 15.08 (3.16) |  | 6.24 (1.80) |  |
| Yin 2016 | 49.5 (8.72) | | 27.0 (1.08) | 27.2 (0.96) | 14.6 (2.18) | 14.2 (2.55) | 5.91 (0.27) | 5.75 (0.3) |
| Salekzamani 2016 | 40.49 (5.04) | | 33.17 (4.83) | 33.58 (4.35) | None | | 5.16 (0.83) | 5.27 (0.67) |
| Mahmood 2017 | 47.7 (8.97) | 46.6 (8.51) | 29.1 (4.06) | 29.7 (4.44) | 15.4 (9.03) | 13.3 (7.91) | 5.72 (0.88) | 5.72 (1.38) |
| Makariou 2017 | 52 (9.00) | 51 (12.00) | 31.0 (5.00) | 33.4 (6.00) | 16.79 (8.15) | 13.11 (9.06) | 5.72 (0.83) | 5.38 (0.61) |
| Makariou 2018 | 53 (7.00) | 52 (15.00) | 31.2 (5.30) | 33.4 (6.00) | 16.92 (8.09) | 13.04 (9.06) | None | |
| Farag 2018 | 40.4 (5.90) | 41.6 (6.40) | 33.4 (4.30) | 30.1 (4.70) | 10.4 (3.20) | 11 (4.00) | 5.88 (0.66) | 5.99 (0.95) |
|  | 40.5 (5.90) | 42.6 (5.60) | 33.1 (5.90) | 32.8 (4.30) | 10.8 (2.80) | 12.2 (4.00) | 5.89 (1.01) | 6.14 (0.96) |
| Mohammadi 2018 | 45.4 (8.9) | 45.6 (8.7) | 30.1 (2.6) | 30.8 (2.2) | 26.04 (13.96) | 26.08 (13.04) | 5.54 (0.94) | 5.42 (1.01) |
| Farag 2019 | 40.42 (5.89) | 42.6 (5.62) | 33.43 (4.26) | 32.81 (4.32) | 10.42 (3.24) | 12.16 (3.95) | 5.89 (0.66) | 6.14 (0.96) |
|  | 40.54 (5.94) |  | 33.12 (5.92) |  | 10.75 (2.79) |  | 5.99 (0.95) |  |
| Ferreira 2019 | 58.8 (6.60) | 59.3 (6.70) | 29.4 (5.4) | 29.9 (4.7) | 15 (7.50) | 16.9 (6.70) | 5.13 (0.59) | 5.19 (0.60) |
| Wallace 2019 | 52.4 (2.00) | 54.0 (1.70) | 34.7 (8.00) | 33.9 (5.60) | 12.28 (5.72) | 12 (6.16) | 6 (0.50) | 6.2 (0.70) |
| Bhatt 2020 | 20-60 | | 31.1 (6.20) | 28.8 (3.90) | 11.96 (2.36) | 12.84 (2.08) | 6.21 (0.39) | 6.18 (0.42) |
| Taghizadeh 2021 | 41.6 (8.26) | | 24.61 (3.76) | 24.46 (3.49) | 14.47 (6.07) | 15.25 (5.82) | None | |
| Sharifan 2021 | 43.47 (7.21) | 43.19 (7.21) | 23.8 (3.65) | 23.27 (3.27) | 14.14 (5.04) | 15.34 (5.68) | 5.48 (1.30) | 5.28 (1.05) |
|  | 40.42 (8.03) | 40.26 (8.27) | 22.95 (2.96) | 23.11 (3.19) | 14.08 (5.15) | 14.02 (5.16) | 5.47 (0.82) | 5.43 (1.02) |
| Najafi 2022 | 56.01 (2.23) | 45.25 (2.22) | 34.51 (1.21) | 33.94 (1.24) | 10~20 | | 7.96 (0.07) | 8.04 (0.08) |
|  | 55.25 (2.01) | 56.36 (1.91) | 34.82 (1.20) | 34.41 (1.01) |  |  | 8.05 (0.10) | 8.07 (0.08) |

| **Author & year** | **TC (mmol/L)** | | **LDL-C (mmol/dL)** | | **HDL-C (mmol/L)** | | **TG (mmol/L)** | |
| --- | --- | --- | --- | --- | --- | --- | --- | --- |
|  | **Intervention** | **Control** | **Intervention** | **Control** | **Intervention** | **Control** | **Intervention** | **Control** |
| Jorde 2010 | 5.43 (1.06) | 5.29 (0.98) | 3.91 (1.02) | 3.79 (0.92) | 1.37 (0.32) | 1.37 (0.39) | 1.53 (0.87) | 1.49 (0.77) |
|  | 5.32 (0.96) |  | 3.76 (0.91) |  | 1.41 (0.36) |  | 1.57 (0.98) |  |
| Kelishadi 2013 | 4.18 (0.08) | 4.25 (0.13) | 2.51 (0.11) | 2.48 (0.07) | 1.22 (0.10) | 1.26 (0.11) | 1.59 (0.27) | 1.62 (0.26) |
| Wong 2013 | 4.67 (0.89) | 4.51 (1.01) | 2.77 (0.71) | 2.66 (0.76) | 1.38 (0.32) | 1.38 (0.33) | 1.57 (0.69) | 1.46 (0.68) |
|  | 4.32 (0.54) |  | 2.50 (0.52) |  | 1.36 (0.31) |  | 1.49 (0.70) |  |
| Yin 2016 | None | | 3.26 (0.25) | 3.2 (0.2) | 1.07 (0.08) | 0.99 (0.07) | 3.34 (0.68) | 3.17 (0.4) |
| Salekzamani 2016 | 5.49 (1.09) | 5.18 (1.02) | 2.95 (0.86) | 3.03 (0.73) | 1.17 (0.21) | 1.17 (0.26) | 3.04 (1.10) | 2.09 (0.69) |
| Makariou 2017 | None | | 3.63 (0.91) | 3.81 (0.67) | 1.24 (0.26) | 1.30 (0.23) | 1.83 (0.81) | 1.84 (0.73) |
| Farag 2018 | 5.04 (0.83) | 3.86 (0.93) | 3.88 (0.93) | 3.13 (1.67) | 1.06 (0.37) | 0.90 (0.45) | 2.08 (1.11) | 2.59 (1.29) |
|  | 4.49 (1.58) | 4.82 (1.01) | 3.98 (1.00) | 3.90 (1.03) | 1.06 (0.43) | 0.78 (0.22) | 1.83 (0.79) | 1.66 (0.49) |
| Mohammadi 2018 | 5.06 (1.40) | 4.94 (0.76) | 2.93 (0.90) | 3.00 (0.74) | 1.06 (0.22) | 1.08 (0.17) | 1.94 (0.85) | 1.96 (0.82) |
| Farag 2019 | 5.04 (0.83) | 4.82 (1.01) | 3.88 (0.93) | 3.90 (1.03) | 1.06 (0.37) | 0.78 (0.22) | 2.08 (1.11) | 1.66 (0.49) |
|  | 4.49 (1.58) |  | 3.13 (1.67) |  | 0.90 (0.45) |  | 2.59 (1.29) |  |
| Ferreira 2019 | 5.47 (1.26) | 5.44 (1.03) | 3.34 (1.03) | 3.36 (0.91) | 1.33 (0.32) | 1.31 (0.32) | 1.76 (1.05) | 1.87 (0.80) |
| Wallace 2019 | 5 (1.10) | 5.1 (1.10) | 2.9 (0.80) | 3 (1.00) | 1.3 (0.30) | 1.3 (0.30) | None | |
| Bhatt 2020 | 4.72 (1.11) | 4.89 (1.13) | 2.91 (0.89) | 3.13 (0.80) | 1.09 (0.30) | 1.10 (0.31) | 1.63 (0.85) | 1.76 (0.68) |
| Sharifan 2021 | 4.98 (0.93) | 5.07 (1.02) | 2.82 (0.69) | 2.94 (0.75) | 1.19 (0.20) | 1.20 (0.23) | 1.56 (0.91) | 1.61 (0.87) |
|  | 5.09 (1.10) | 5.13 (1.01) | 3.00 (0.75) | 3.02 (0.78) | 1.22 (0.23) | 1.18 (0.20) | 1.50 (0.85) | 1.41 (0.68) |
| Najafi 2022 | None | | | | 0.86 (0.05) | 0.88 (0.05) | 2.16 (0.03) | 2.18 (0.03) |
|  |  |  |  |  | 0.91 (0.04) | 0.89 (0.05) | 2.17 (0.03) | 2.17 (0.02) |

| **Author & year** | **Systolic (mmHg)** | | **Diastolic (mmHg)** | | **Fasting insulin (µU/mL)** | | **HOMA-IR** | |
| --- | --- | --- | --- | --- | --- | --- | --- | --- |
|  | **Intervention** | **Control** | **Intervention** | **Control** | **Intervention** | **Control** | **Intervention** | **Control** |
| Jorde 2010 | 124 (15.00) | 125 (16.00) | 76.5 (9.80) | 74.8 (10.00) | 18.21 (9.95) | 18.51 (9.95) | 4.74 (2.86) | 4.9 (2.85) |
|  | 121 (13.00) |  | 74.9 (9.50) |  | 16.52 (7.65) |  | 4.27 (2.19) |  |
| Kelishadi 2013 | None | | | | 14.27 (1.32) | 14.19 (1.20) | 3.21 (0.11) | 3.15 (0.26) |
| Wong 2013 | 134.32 (17.03) | 136.46 (14.99) | 76.57 (12.62) | 80.25 (23.18) | 6.86 (4.61) | 4.78 (3.47) | 2.05 (1.55) | 1.32 (1.03) |
|  | 135.71 (14.79) |  | 78.54 (11.47) |  | 6.35 (4.34) |  | 1.66 (1.03) |  |
| Yin 2016 | 142 (4.86) | 139 (5.52) | 90.1 (3.86) | 88.9 (4.64) | 14.1 (12.5) | 13.2 (11.5) | 4.73 (1.15) | 3.96 (1.72) |
| Salekzamani 2016 | 133 (14.00) | 130 (10.00) | 85 (10.00) | 83 (8.00) | 12.05 (5.19) | 12.75 (4.87) | 2.76 (1.26) | 2.96 (1.19) |
| Mahmood 2017 | 134 (14.9) | 128 (11.4) | 88.1 (9.17) | 84.8 (6.73) | 10.8 (5.14) | 10.7 (4.81) | 2.72 (1.33) | 2.82 (2.01) |
| Makariou 2017 | 134 (14.00) | 132 (13.00) | 85 (6.00) | 85 (9.00) | 11.11 (3.51) | 9.65 (4.53) | 2.76 (1.58) | 2.72 (0.79) |
| Farag 2018 | 129 (12.7) | 128.6 (10.8) | 83.6 (9.4) | 82.5 (5.9) | None | | | |
|  | 127.1 (11.5) | 125.6 (14.5) | 79.8 (9.5) | 80 (6.9) |  |  |  |  |
| Mohammadi 2018 | 132.5 (15.50) | 129.4 (16.60) | 90.7 (9.50) | 89.0 (9.10) | 19.8 (8.00) | 17.8 (6.90) | 5.0 (2.50) | 4.7 (2.30) |
| Farag 2019 | 129.04 (12.71) | 125.6 (14.52) | 83.57 (9.37) | 80 (6.92) | None | | | |
|  | 127.08 (11.5) |  | 79.79 (9.49) |  |  |  |  |  |
| Ferreira 2019 | 134.3 (19.80) | 136.5 (21.00) | 81.5 (12.60) | 81 (10.80) | 11.7 (8.10) | 10.1 (5.80) | 2.8 (2.10) | 2.4 (1.50) |
| Wallace 2019 | 133 (11.00) | 135 (16.00) | 82 (8.00) | 82 (7.00) | 14.8 (9.70) | 16.7 (7.40) | 4 (3.00) | 4.7 (2.30) |
| Bhatt 2020 | None | | | | 14.2 (9.40) | 11.4 (7.50) | 1.39 (0.08) | 1.81 (0.09) |
| Sharifan 2021 | 123.74 (17.20) | 121.31 (18.01) | 80.2 (12.17) | 76.14 (13.14) | 20.12 (10.45) | 10.87 (4.42) | 5.08 (3.10) | 2.71 (1.48) |
|  | 117.39 (12.48) | 120.63 (16.78) | 75.81 (10.58) | 76.98 (11.02) | 12.03 (4.54) | 18.16 (4.77) | 2.88 (1.04) | 4.38 (1.26) |

NCEP-ATP Ⅲ criteria: National Cholesterol Education Program Adult Treatment Panel III; IDF criteria: International Diabetes Federation; ADA criteria: American Diabetes Association; MetS: metabolic syndrome; PA: physical activities; AT: aerobic training; BMI: body mass index, FPG: fasting plasma glucose, TC: total cholesterol, LDL-C: low density lipoprotein cholesterol, HDL-C: high density lipoprotein cholesterol, TG: triglyceride, HOMA-IR: homeostasis model assessment of insulin resistance.

## 2.2. Second outcomes

***2.2.1. Effect of VD supplementation on serum 25(OH)D.*** The test for heterogeneity (I² = 97%, P < 0.00001) of the 20 RCTs (Figure 3H) suggested a firm heterogeneity among the studies. We eliminated these studies one by one through a sensitivity analysis and found that the studies did not significantly alter the overall effect size. As a result, a randomized model was used to combine the effect sizes. The result showed that VD intervention significantly improved serum 25(OH)D levels compared to the control groups [MD = 13.51, 95% CI (10.55, 16.46), P < 0.00001] mg/dL.

**Table S6.** Diagnostic criteria for serum 25(OH)D.

| Serum 25(OH)D (ng/mL) | deficiency | insufficiency | sufficiency |
| --- | --- | --- | --- |
| Endocrine Society clinical practice guideline | <20 | 20~30 | >30 |
| The Global Consensus recommended classifying VD | <12 | 12~20 | >20 |

***2.2.2. Effect of VD supplementation on biomarkers of blood pressure.*** The heterogeneity test for systolic blood pressure (I² = 61%, P = 0.003) of the 12 RCTs (Figure S1A) suggested a moderate heterogeneity among the studies. After excluding Jorde (2) et al. (2010) [13] by sensitivity analysis, a fixed-effects model (I² = 43%, P = 0.06) was used to combine effect sizes and showed that VD supplementation significantly reduced systolic blood pressure [MD = -1.22, 95% CI (-2.40, -0.04), P = 0.04] mmHg. In Figure S1B, there was no heterogeneity in diastolic blood pressure (I² = 0%, P = 0.85), so a fixed-effects model was used to combine effect sizes. The results showed that VD supplementation did not significantly improve diastolic blood pressure [MD = -0.51, 95% CI (-1.31, 0.29), P = 0.21] mmHg.

***2.2.3. Effect of VD supplementation on biomarkers of obesity.***

***Waist circumference, WC.*** The test for heterogeneity (I² = 54%, P = 0.007) of the 15 RCTs (Figure S1C) in anthropometrics suggested that there was a moderate heterogeneity among studies. After excluding Najafi (2) et al. (2022) [29] by sensitivity analysis and using a fixed model (I² = 0%, P = 0.95) to combine effect sizes, we found that VD supplementation did not significantly improve WC [MD = -0.57, 95% CI (-1.29, 0.16), P = 0.12) P = 0.12] cm.

***Body mass index, BMI.*** The test for heterogeneity (I² = 0%, P = 0.65) regarding the change in BMI (Figure S1D) suggested that there was no heterogeneity among studies. The results showed no statistically significant change in BMI for the VD intervention compared to the control groups MD = -0.14, 95% CI (-0.31, 0.03), P = 0.11] kg/m^2^.





**Figure S1.** Forest plot for fixed-effects model of the effect of VD supplementation on outcome indicators for blood pressure **(A-B)** and obesity **(C-D).** Abbreviations: WC, waist circumference; BMI, body mass index.

## 2.3. Sensitivity analysis and publication bias

The Egger regression test was employed to assess publication bias in 20 RCTs regarding serum 25(OH)D levels. The results revealed a significant deviation of the intercept term in the Egger regression test (intercept = 13.50, p < 0.05) from zero, indicating the presence of publication bias (Figure S2A-B). Consequently, we utilized the Trim and Fill method to estimate and rectify potential bias. Using this method, we estimated the number of studies that might have been omitted on the left-hand side to be zero (Figure S2C), suggesting that in the current dataset, there was insufficient evidence to suggest that the presence of unpublished studies had an impact on the estimation of the combined effect.


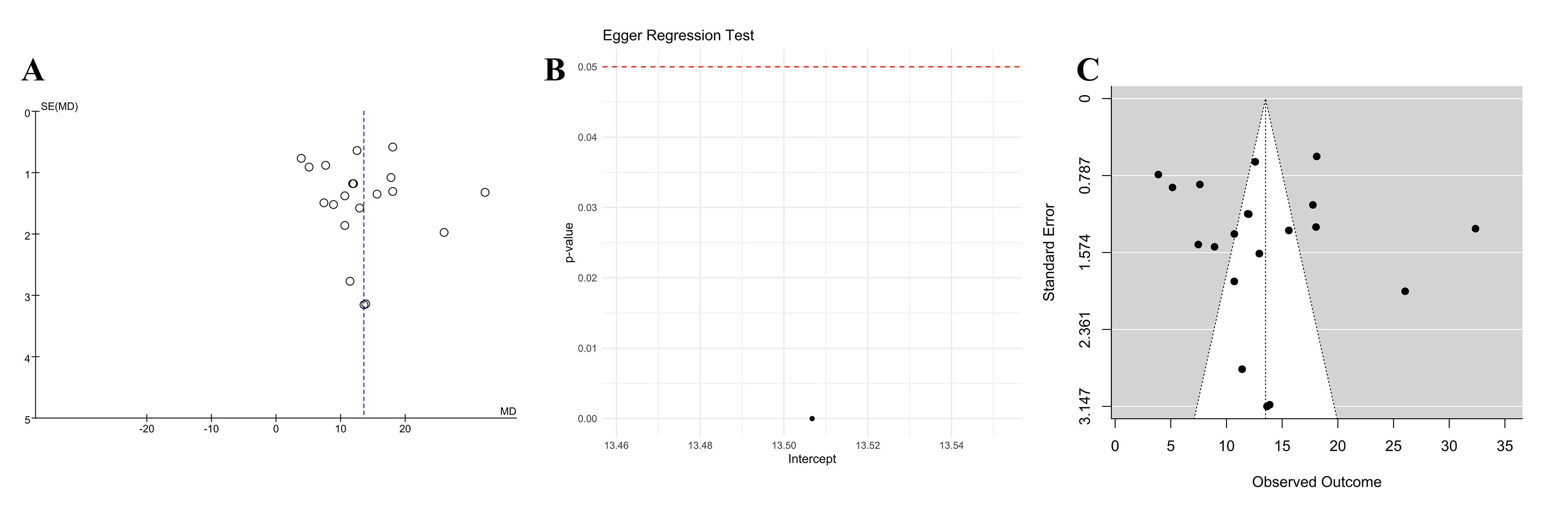


**Figure S2.** The Egger regression test for bias in 20 RCTs on serum 25(OH)D levels.

In addition, we found that the funnel plots of lipids, insulin resistance, blood pressure, and anthropometry-related metrics (Figure S3) were largely symmetrical without significant publication bias after excluding outliers through sensitivity analysis.


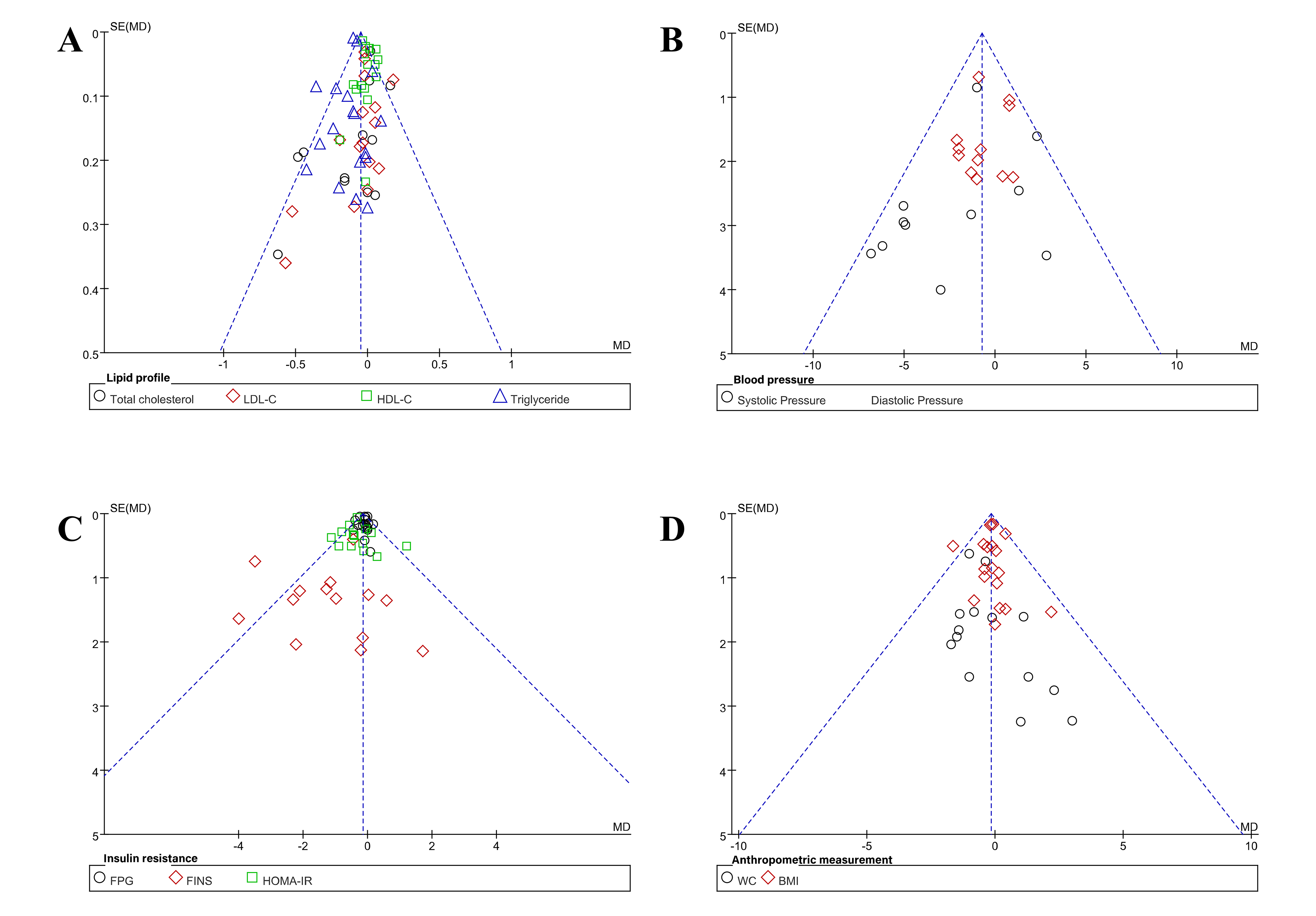


**Figure S3.** Funnel plot of VD supplementation on lipids profile **(A)**, insulin resistance **(B)**, blood pressure **(C)** and anthropometry-related **(D)** outcome indicators in subjects.

## 2.4. Molecular dynamics simulations

To obtain the best initial conformation of the protein-ligand as possible, we performed semi-flexible docking of the active sites of the proteins through the genetic algorithm module of AutoDock-4.2, and the resulting complex conformation was used for molecular dynamics simulations. To verify the reliability of the docking results, we used AutoDock-4.2 to redock the proto-ligands of the three proteins. In Table S7, the results showed that the RMSD of the original co-crystallized ligand backbone stacking was less than 2 Å for all of them, indicating that the docking method is suitable for the system we need to study.

**Table S7.** The result of molecular docking through AutoDock-4.2.

| **Core Gene** | **PDB** | **Resolution (Å)** | **Redocking (Å)** | **Dock Score (kcal/mol)** | |
| --- | --- | --- | --- | --- | --- |
|  |  |  |  | **VD_2_** | **VD_3_** |
| PPARG | 6FZG | 2.10 | OL: EDK*, RMSD = 1.44 | -11.65 | -10.9 |
| FABP4 | 5EDB | 1.18 | OL: 5M8*, RMSD = 0.00 | -9.18 | -8.69 |
| HMGCR | 1HW8 | 2.10 | OL: 114*, RMSD = 1.01 | -8.04 | -6.55 |


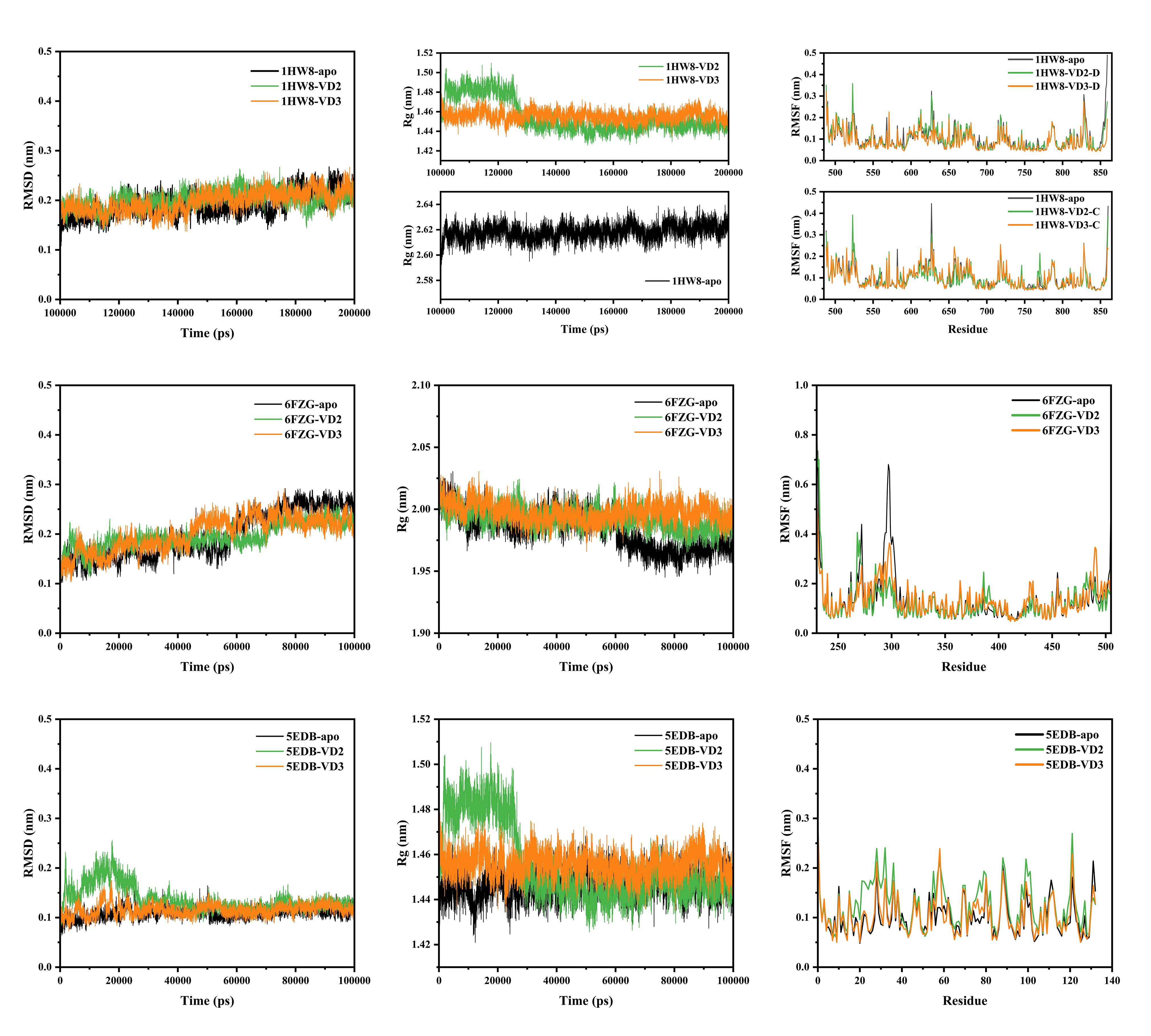


**Figure S4.** The results of molecular dynamics simulations. RMSD **(A1, B1, C1)**, Rg **(A2, B2, C2)**, RMSF **(A3, B3, C3)** of three proteins in free and combined states with VD. The simulation time was 100 ns for all systems except for the complex 1HW8-VD system, which was 200 ns.


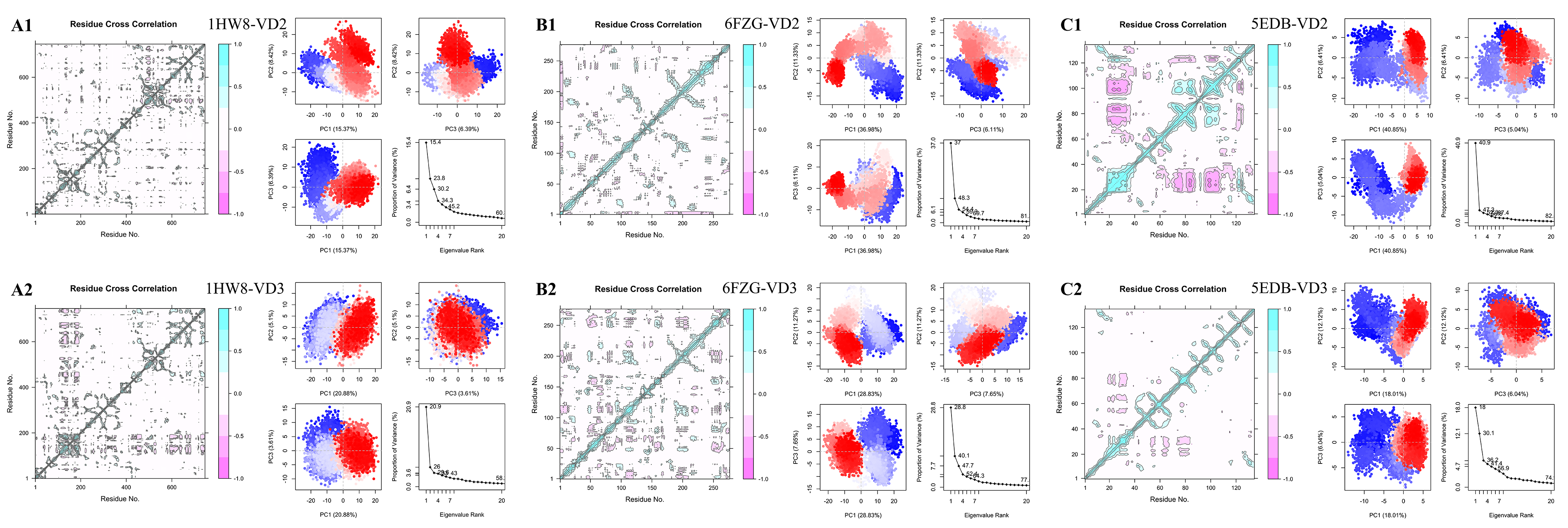


**Figure S5.** Plot of the co-correlation matrix with principal component analysis for the six complex systems.





**Figure S6.** Free energy landscape of six protein-ligand complex systems.


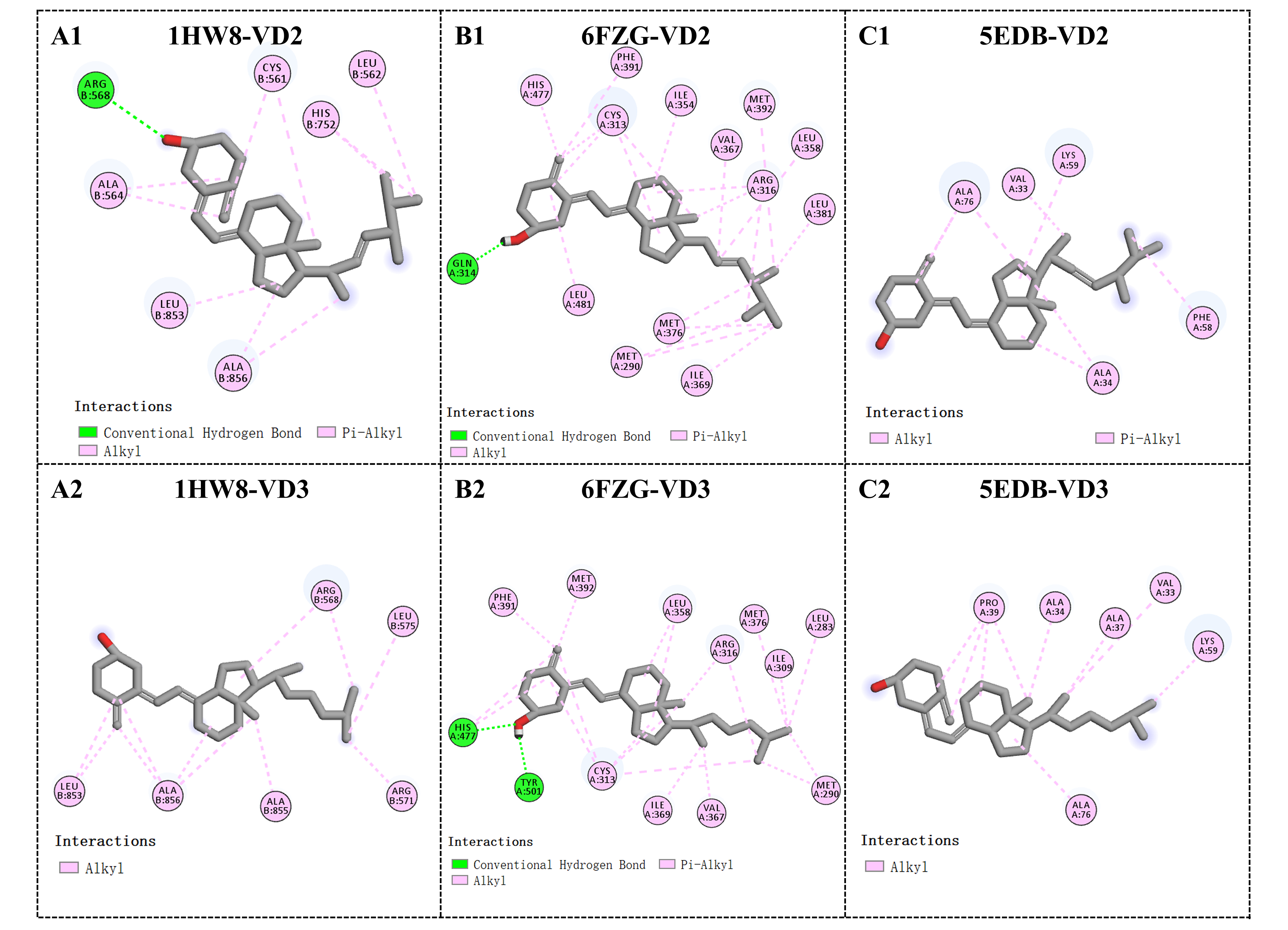


**Figure S7.** Predicting protein-ligand 2D interactions diagrams through *Discovery Studio 2019.*


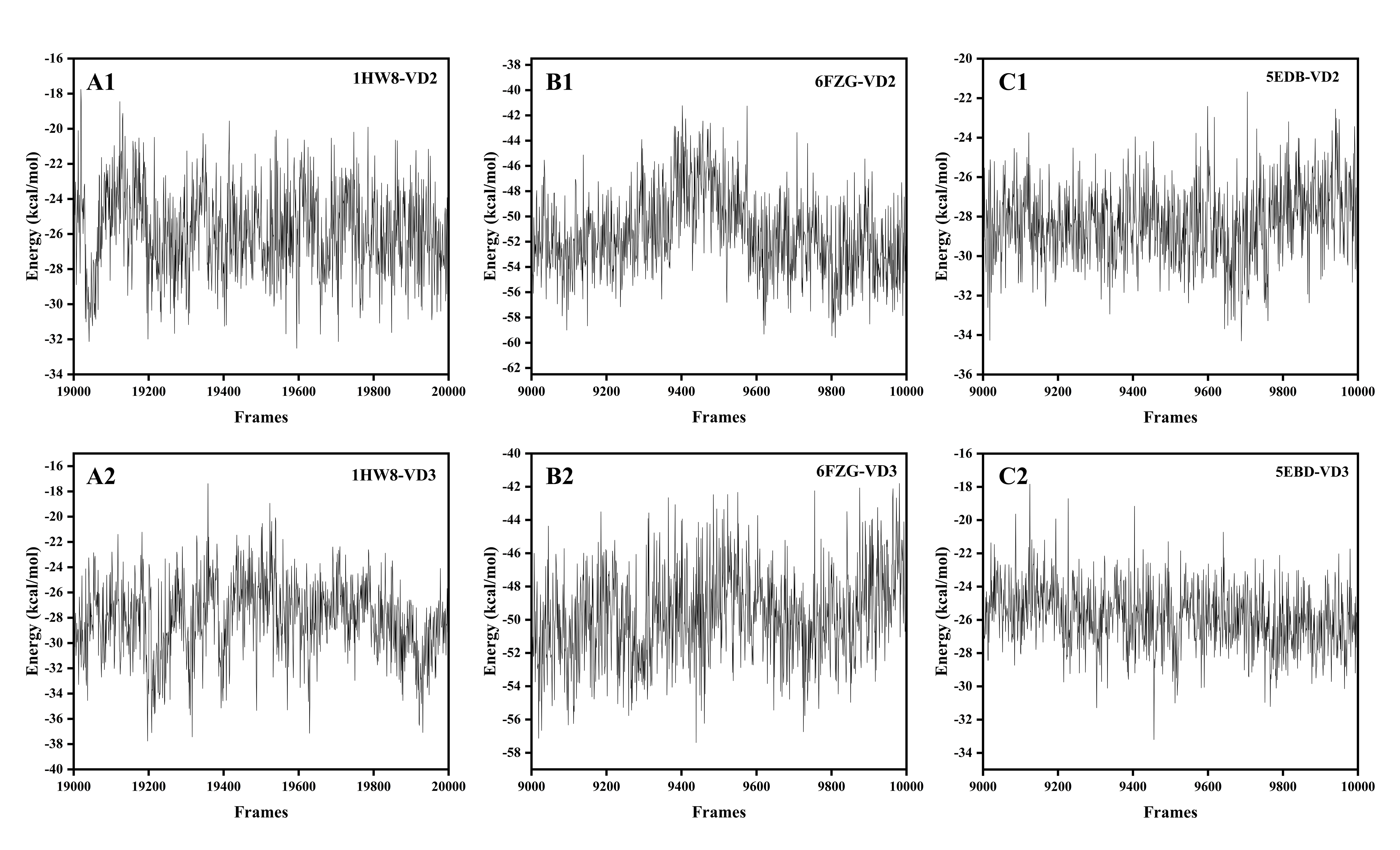


**Figure S8.** Calculation of binding free energies for six complex systems using the MM-GBSA method.


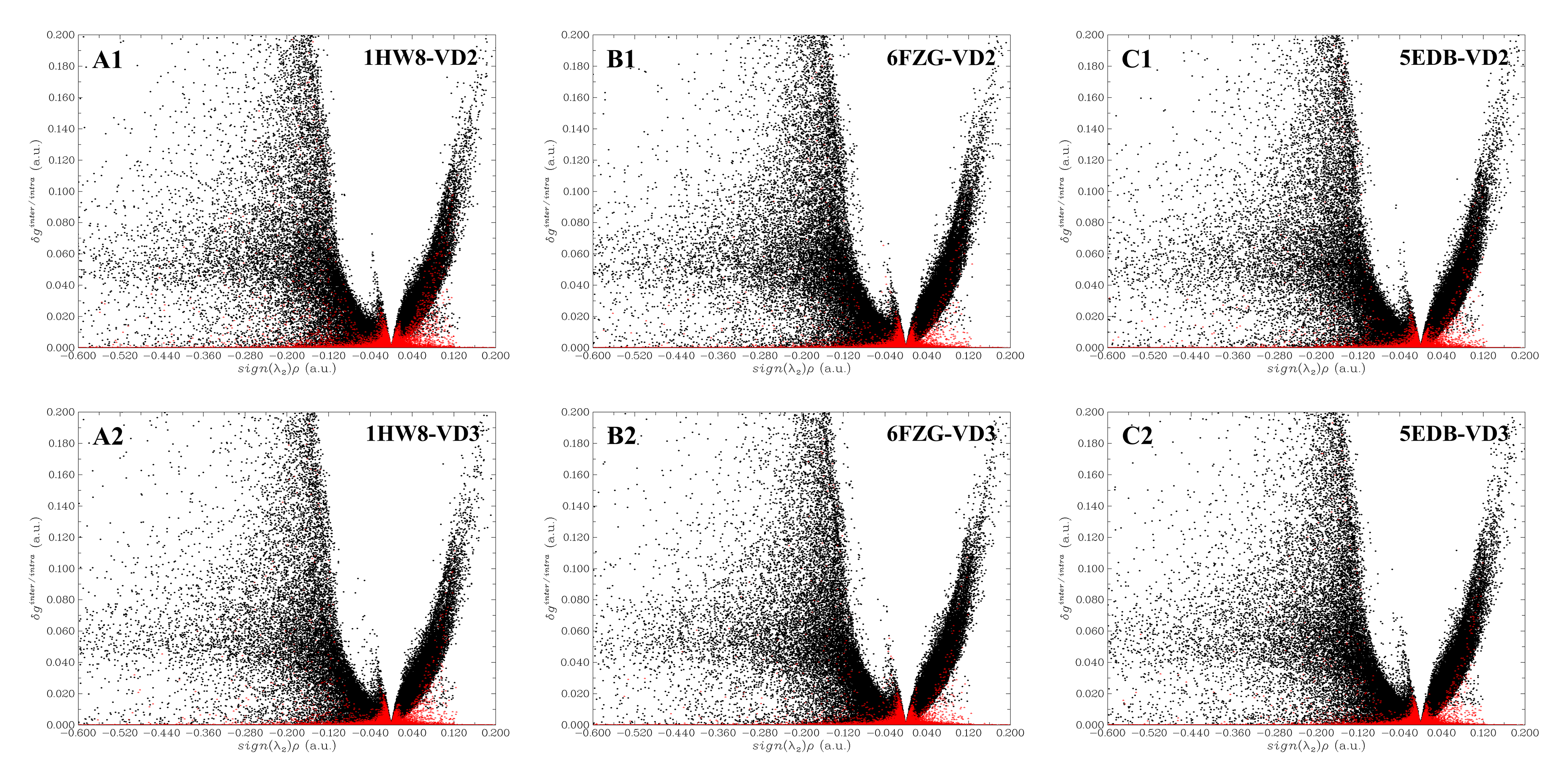


**Figure S9.** Visual scatter maps of δ_g_ ^inter^ and δ_g_ ^intra^ vs. sign(λ_2_)*p* in independent gradient model.

**References**

1. Moher, D.; Liberati, A.; Tetzlaff, J.; Altman, D. G.; Group*, P., Preferred reporting items for systematic reviews and meta-analyses: the PRISMA statement. *Annals of internal medicine* **2009,** *151* (4), 264-269, https://doi.org/10.7326/0003-4819-151-4-200908180-00135.

2. Piñero, J.; Bravo, À.; Queralt-Rosinach, N.; Gutiérrez-Sacristán, A.; Deu-Pons, J.; Centeno, E.; García-García, J.; Sanz, F.; Furlong, L. I., DisGeNET: a comprehensive platform integrating information on human disease-associated genes and variants. *Nucleic Acids Research* **2017,** *45* (D1), D833-D839, https://doi.org/10.1093/nar/gkw943.

3. Stelzer, G.; Rosen, N.; Plaschkes, I.; Zimmerman, S.; Twik, M.; Fishilevich, S.; Stein, T. I.; Nudel, R.; Lieder, I.; Mazor, Y.; Kaplan, S.; Dahary, D.; Warshawsky, D.; Guan-Golan, Y.; Kohn, A.; Rappaport, N.; Safran, M.; Lancet, D., The GeneCards Suite: From Gene Data Mining to Disease Genome Sequence Analyses. *Current Protocols in Bioinformatics* **2016,** *54* (1), 1.30.1-1.30.33, https://doi.org/10.1002/cpbi.5.

4. Amberger, J. S.; Bocchini, C. A.; Schiettecatte, F.; Scott, A. F.; Hamosh, A., OMIM.org: Online Mendelian Inheritance in Man (OMIM®), an online catalog of human genes and genetic disorders. *Nucleic Acids Research* **2015,** *43* (D1), D789-D798, https://doi.org/10.1093/nar/gku1205.

5. Wishart, D. S.; Feunang, Y. D.; Guo, A. C.; Lo, E. J.; Marcu, A.; Grant, J. R.; Sajed, T.; Johnson, D.; Li, C.; Sayeeda, Z.; Assempour, N.; Iynkkaran, I.; Liu, Y.; Maciejewski, A.; Gale, N.; Wilson, A.; Chin, L.; Cummings, R.; Le, D.; Pon, A.; Knox, C.; Wilson, M., DrugBank 5.0: a major update to the DrugBank database for 2018. *Nucleic Acids Research* **2018,** *46* (D1), D1074-D1082, https://doi.org/10.1093/nar/gkx1037.

6. Whirl-Carrillo, M.; Huddart, R.; Gong, L.; Sangkuhl, K.; Thorn, C. F.; Whaley, R.; Klein, T. E., An Evidence-Based Framework for Evaluating Pharmacogenomics Knowledge for Personalized Medicine. *Clinical Pharmacology & Therapeutics* **2021,** *110* (3), 563-572, https://doi.org/10.1002/cpt.2350.

7. Zhou, Y.; Zhang, Y.; Lian, X.; Li, F.; Wang, C.; Zhu, F.; Qiu, Y.; Chen, Y., Therapeutic target database update 2022: facilitating drug discovery with enriched comparative data of targeted agents. *Nucleic Acids Research* **2022,** *50* (D1), D1398-D1407, https://doi.org/10.1093/nar/gkab953.

8. Wang, X.; Shen, Y.; Wang, S.; Li, S.; Zhang, W.; Liu, X.; Lai, L.; Pei, J.; Li, H., PharmMapper 2017 update: a web server for potential drug target identification with a comprehensive target pharmacophore database. *Nucleic Acids Research* **2017,** *45* (W1), W356-W360, https://doi.org/10.1093/nar/gkx374.

9. Daina, A.; Michielin, O.; Zoete, V., SwissTargetPrediction: updated data and new features for efficient prediction of protein targets of small molecules. *Nucleic Acids Research* **2019,** *47* (W1), W357-W364, https://doi.org/10.1093/nar/gkz382.

10. Keiser, M. J.; Roth, B. L.; Armbruster, B. N.; Ernsberger, P.; Irwin, J. J.; Shoichet, B. K., Relating protein pharmacology by ligand chemistry. *Nature Biotechnology* **2007,** *25* (2), 197-206, https://doi.org/10.1038/nbt1284.

11. Gallo, K.; Goede, A.; Preissner, R.; Gohlke, B. O., SuperPred 3.0: drug classification and target prediction—a machine learning approach. *Nucleic Acids Research* **2022,** *50* (W1), W726-W731, https://doi.org/10.1093/nar/gkac297.

12. Yang, J.; Kwon, S.; Bae, S.; Park, K. M.; Yoon, C.; Lee, J.; Seok, C., GalaxySagittarius: Structure- and Similarity-Based Prediction of Protein Targets for Druglike Compounds. *Journal of Chemical Information and Modeling* **2020,** *60* (6), 3246-3254, https://doi.org/10.1021/acs.jcim.0c00104.

13. Jorde, R.; Sneve, M.; Torjesen, P.; Figenschau, Y., No improvement in cardiovascular risk factors in overweight and obese subjects after supplementation with vitamin D3 for 1 year. *Journal of Internal Medicine* **2010,** *267* (5), 462-472, https://doi.org/10.1111/j.1365-2796.2009.02181.x.

14. Kelishadi, R.; Salek, S.; Salek, M.; Hashemipour, M.; Movahedian, M., Effects of vitamin D supplementation on insulin resistance and cardiometabolic risk factors in children with metabolic syndrome: a triple-masked controlled trial. *Jornal de Pediatria* **2013,** *90*, https://doi.org/10.1016/j.jped.2013.06.006.

15. Wongwiwatthananukit, S.; Sansanayudh, N.; Phetkrajaysang, N.; Krittiyanunt, S., Effects of vitamin D2 supplementation on insulin sensitivity and metabolic parameters in metabolic syndrome patients. *Journal of Endocrinological Investigation* **2013,** *36* (8), 558-563, https://doi.org/10.3275/8817.

16. Yin, X.; Yan, L.; Lu, Y.; Jiang, Q.; Pu, Y.; Sun, Q., Correction of hypovitaminosis D does not improve the metabolic syndrome risk profile in a Chinese population: a randomized controlled trial for 1 year. *Asia Pacific journal of clinical nutrition* **2016,** *25* (1), 71-77, https://doi.org/10.3316/ielapa.908305579873545.

17. Salekzamani, S.; Mehralizadeh, H.; Ghezel, A.; Salekzamani, Y.; Jafarabadi, M. A.; Bavil, A. S.; Gargari, B. P., Effect of high-dose vitamin D supplementation on cardiometabolic risk factors in subjects with metabolic syndrome: a randomized controlled double-blind clinical trial. *Journal of Endocrinological Investigation* **2016,** *39* (11), 1303-1313, https://doi.org/10.1007/s40618-016-0507-8.

18. Mahmood, S. F.; Idiculla, J.; Joshi, R.; Joshi, S.; Kulkarni, S., Vitamin D Supplementation in Adults with Vitamin D Deficiency and Its Effect on Metabolic Syndrome – A Randomized Controlled Study. *International Journal for Vitamin and Nutrition Research* **2017,** *86* (3-4), 121-126, https://doi.org/10.1024/0300-9831/a000426.

19. Makariou, S. E.; Elisaf, M.; Challa, A.; Tentolouris, N.; Liberopoulos, E. N., No effect of vitamin D supplementation on cardiovascular risk factors in subjects with metabolic syndrome: a pilot randomised study. *Arch Med Sci Atheroscler Dis* **2017,** *2* (1), 52-60, https://doi.org/10.5114/amsad.2017.70504.

20. Makariou, S. E.; Elisaf, M.; Challa, A.; Tellis, C.; Tselepis, A. D.; Liberopoulos, E. N., Effect of combined vitamin D administration plus dietary intervention on oxidative stress markers in patients with metabolic syndrome: A pilot randomized study. *Clinical Nutrition ESPEN* **2018,** *29*, 198-202, https://doi.org/10.1016/j.clnesp.2018.10.004.

21. Farag, H. A. M.; Hosseinzadeh-Attar, M. J.; Muhammad, B. A.; Esmaillzadeh, A.; Bilbeisi, A. H. E., Comparative effects of vitamin D and vitamin C supplementations with and without endurance physical activity on metabolic syndrome patients: a randomized controlled trial. *Diabetology & Metabolic Syndrome* **2018,** *10* (1), 80, https://doi.org/10.1186/s13098-018-0384-8.

22. Mohammadi-Sartang, M.; Bellissimo, N.; Totosy de Zepetnek, J. O.; Brett, N. R.; Mazloomi, S. M.; Fararouie, M.; Bedeltavana, A.; Famouri, M.; Mazloom, Z., The effect of daily fortified yogurt consumption on weight loss in adults with metabolic syndrome: A 10-week randomized controlled trial. *Nutrition, Metabolism and Cardiovascular Diseases* **2018,** *28* (6), 565-574, https://doi.org/10.1016/j.numecd.2018.03.001.

23. Farag, H. A. M.; Hosseinzadeh-Attar, M. J.; Muhammad, B. A.; Esmaillzadeh, A.; Hamid el Bilbeisi, A., Effects of vitamin D supplementation along with endurance physical activity on lipid profile in metabolic syndrome patients: A randomized controlled trial. *Diabetes & Metabolic Syndrome: Clinical Research & Reviews* **2019,** *13* (2), 1093-1098, https://doi.org/10.1016/j.dsx.2019.01.029.

24. Ferreira, P. P.; Cangussu, L.; Bueloni-Dias, F. N.; Orsatti, C. L.; Schmitt, E. B.; Nahas-Neto, J.; Nahas, E. A. P., Vitamin D supplementation improves the metabolic syndrome risk profile in postmenopausal women. *Climacteric* **2019,** *23* (1), 24-31, https://doi.org/10.1080/13697137.2019.1611761.

25. Wallace, H. J.; Holmes, L.; Ennis, C. N.; Cardwell, C. R.; Woodside, J. V.; Young, I. S.; Bell, P. M.; Hunter, S. J.; McKinley, M. C., Effect of vitamin D3 supplementation on insulin resistance and β-cell function in prediabetes: a double-blind, randomized, placebo-controlled trial. *The American journal of clinical nutrition* **2019,** *110* (5), 1138-1147, https://doi.org/10.1093/ajcn/nqz171.

26. Bhatt, S. P.; Misra, A.; Pandey, R. M.; Upadhyay, A. D.; Gulati, S.; Singh, N., Vitamin D Supplementation in Overweight/obese Asian Indian Women with Prediabetes Reduces Glycemic Measures and Truncal Subcutaneous Fat: A 78 Weeks Randomized Placebo-Controlled Trial (PREVENT-WIN Trial). *Scientific Reports* **2020,** *10* (1), 220, https://doi.org/10.1038/s41598-019-56904-y.

27. Taghizadeh, N.; Sharifan, P.; Ekhteraee Toosi, M. S.; Najar Sedgh Doust, F.; Darroudi, S.; Afshari, A.; Rezaie, M.; Safarian, M.; Vatanparast, H.; Eslami, S.; Ghazizadeh, H.; Khorasanchi, Z.; Bagherniya, M.; Ferns, G.; Assaran Darban, R.; Ghayour-Mobarhan, M., The effects of consuming a low-fat yogurt fortified with nano encapsulated vitamin D on serum pro-oxidant-antioxidant balance (PAB) in adults with metabolic syndrome; a randomized control trial. *Diabetes & Metabolic Syndrome: Clinical Research & Reviews* **2021,** *15* (6), 102332, https://doi.org/10.1016/j.dsx.2021.102332.

28. Sharifan, P.; Ziaee, A.; Darroudi, S.; Rezaie, M.; Safarian, M.; Eslami, S.; Khadem-Rezaiyan, M.; Tayefi, M.; Mohammadi Bajgiran, M.; Ghazizadeh, H.; Khorasanchi, Z.; Bagherniya, M.; Sardar, M. A.; Ferns, G.; Vatanparast, H.; Ghayour Mobarhan, M., Effect of low-fat dairy products fortified with 1500IU nano encapsulated vitamin D3 on cardiometabolic indicators in adults with abdominal obesity: a total blinded randomized controlled trial. *Current Medical Research and Opinion* **2021,** *37* (4), 579-588, https://doi.org/10.1080/03007995.2021.1874324.

29. Nazarabadi, P. N.; Etemad, Z.; Hoseini, R.; Moradi, F., Anti-Inflammatory effects of a period of aerobic training and vitamin D supplementation in postmenopausal women with metabolic syndrome. *International Journal of Preventive Medicine* **2022,** *13*, https://doi.org/10.4103/ijpvm.IJPVM_312_20.
